# Acupuncture for the Relief of Chronic Pain: A Synthesis of Systematic Reviews

**DOI:** 10.3390/medicina56010006

**Published:** 2019-12-24

**Authors:** Carole A. Paley, Mark I. Johnson

**Affiliations:** 1 Research and Development Dept, Airedale National Health Service (NHS) Foundation Trust, Skipton Road, Steeton, Keighley BD20 6TD, UK; 2 Centre for Pain Research, School of Clinical and Applied Sciences, Leeds Beckett University, City Campus, Leeds LS1 3HE, UK; M.Johnson@leedsbeckett.ac.uk

**Keywords:** acupuncture, pain, systematic review, evidence synthesis

## Abstract

*Background and Objectives*: It is estimated that 28 million people in the UK live with chronic pain. A biopsychosocial approach to chronic pain is recommended which combines pharmacological interventions with behavioural and non-pharmacological treatments. Acupuncture represents one of a number of non-pharmacological interventions for pain. In the current climate of difficult commissioning decisions and constantly changing national guidance, the quest for strong supporting evidence has never been more important. Although hundreds of systematic reviews (SRs) and meta-analyses have been conducted, most have been inconclusive, and this has created uncertainty in clinical policy and practice. There is a need to bring all the evidence together for different pain conditions. The aim of this review is to synthesise SRs of RCTs evaluating the clinical efficacy of acupuncture to alleviate chronic pain and to consider the quality and adequacy of the evidence, including RCT design. *Materials and Methods*: Electronic databases were searched for English language SRs and meta-analyses on acupuncture for chronic pain. The SRs were scrutinised for methodology, risk of bias and judgement of efficacy. *Results*: A total of 177 reviews of acupuncture from 1989 to 2019 met our eligibility criteria. The majority of SRs found that RCTs of acupuncture had methodological shortcomings, including inadequate statistical power with a high risk of bias. Heterogeneity between RCTs was such that meta-analysis was often inappropriate. *Conclusions*: The large quantity of RCTs on acupuncture for chronic pain contained within systematic reviews provide evidence that is conflicting and inconclusive, due in part to recurring methodological shortcomings of RCTs. We suggest that an enriched enrolment with randomised withdrawal design may overcome some of these methodological shortcomings. It is essential that the quality of evidence is improved so that healthcare providers and commissioners can make informed choices on the interventions which can legitimately be provided to patients living with chronic pain.

## 1. Introduction

The World Health Organisation (WHO) recognises chronic pain as a long-term condition in its own right and as a secondary consequence of other long-term conditions [1]. It has been estimated that 28 million adults in the UK (43%) are affected by chronic pain and that the pain of 7.9 million of these adults is moderately or severely limiting [2]. The prevalence of chronic pain is higher in older age groups, with an estimated 62% of people over 75 being affected [2]. Individuals living with pain often experience a very poor quality of life, it affects their ability to work, socialise, sleep and maintain good relationships and can lead to depressive illness, decreased motivation and a reduction in physical activity [3]. As such, chronic pain represents a major challenge for health service provision and government policy.

Current guidance from the International Association for the Study of Pain (IASP) recommends a biopsychosocial approach to pain utilising a multidisciplinary, multimodal, stepwise approach which combines pharmacological interventions with behavioural and non-pharmacological treatments [4]. Non-pharmacological interventions are recommended as part of a comprehensive pain management programme, including lifestyle adjustments, pain education, and physical, psychological and complementary therapies.

In the UK, acupuncture has been available in some parts of the National Health Service (NHS) for decades as a non-pharmacological intervention to manage acute or chronic pain. In the NHS, acupuncture is administered by Allied Health Professionals, Nurses or Doctors. Outside the NHS, acupuncture is available from a variety of sources, including ‘traditional’ acupuncturists, sports therapists, osteopaths and chiropractors.

Acupuncture is an age-old technique which became part of modern medicine in the 1970s. In modern medicine, traditional forms of acupuncture, based on the ancient Chinese concept of *qi* and meridians, have been superseded by acupuncture based on a neurophysiological model [5,6]. The unique identity of acupuncture lies in the process of inserting needles (‘acu’) in the skin (‘puncture’), although a modern definition should include the need to do this at specific points in accordance with known physiological or anatomical rationale [7].

Over the past two decades, the quantity of clinical studies on the use of acupuncture for various types of pain has significantly increased. In 2013, it was estimated that over 3000 clinical trials had been published [8] with over one hundred systematic reviews (SRs) (some with meta-analyses) attempting to synthesise available evidence. Many SRs of randomised controlled trials (RCTs) of acupuncture have been inconclusive and this has created uncertainty in clinical policy and practice. This uncertainty was highlighted in 2016 when the National Institute for Health and Care Excellence (NICE) reversed its 2009 recommendation to offer acupuncture as a first line treatment for non-specific, chronic low back pain because evidence indicated that it was no more effective than sham acupuncture [9,10,11]. Interestingly, there had been no significant change in evidence provided by RCTs between 2009 and 2016. Presently, NICE only recommends acupuncture as a prophylactic treatment for chronic tension-type headache and migraine [12,13].

In the face of conflicting evidence and continually changing guidance, it is unsurprising that acupuncture practitioners are finding that an intervention that, anecdotally at least, is often well received by patients in the clinic and appears to have good results, is rejected by commissioners and policy makers and regarded in some quarters as a ‘theatrical placebo’ [8,14]. One reason for this uncertainty may be related to the clinical research methodologies used to determine clinical efficacy.

Policy makers give credence to the findings of RCTs because they are the ‘gold standard’ methodology for evaluating clinical efficacy. RCTs enable isolation of the effects (benefit and harm) associated with the active ingredient of a treatment from effects associated with the act of receiving a treatment, i.e., believing that an active ingredient of a treatment has been received. This is operationalised by using needles to puncture the skin at defined points compared with pretending to puncture the skin at defined points (i.e., a ‘placebo’ or ‘sham’ intervention).

Systematic reviews and meta-analyses of multiple RCTs provide an indicator of consistency of findings between RCTs and allow for generalisability of findings [15]. Practitioners and policy makers may feel overwhelmed by the volume of SRs on acupuncture, suggesting a need to bring all this evidence together. In doing so, there is an opportunity to appraise RCT design and whether it is fit for purpose.

The aim of this review is to synthesise evidence from previously published SRs of RCTs evaluating the clinical efficacy of acupuncture to alleviate chronic pain from any source. We have made judgements from a Western medical perspective. Our approach is to outline research findings through commentary rather than a comprehensive objective appraisal of SRs. We appreciate that the non-systematic approach is vulnerable to selection and evaluation biases and opinion-orientated arguments. Nevertheless, our approach enables consideration of issues surrounding the quality and adequacy of the evidence, including RCT design, and provides practitioners and policy makers with a comprehensive source of SRs published to date.

## 2. Materials and Methods

A search of electronic databases (MEDLINE, the Database of Abstracts of Reviews of Effects (DARE) and the Cochrane Library) was conducted in April 2019 and updated in July 2019 using free text search terms ‘acupuncture’, ‘chronic pain’, ‘analgesia’, ‘pain management’, ‘systematic review’ and/or ‘meta-analysis’. The search was restricted to English language databases. Systematic reviews and meta-analyses were screened for eligibility.

### 2.1. Inclusion Criteria

Search results were screened by the authors, CAP and MIJ. All SRs with or without meta-analyses of studies using manual acupuncture, electro-acupuncture, dry needling or auriculotherapy (ear acupuncture) for any chronic pain condition were included. Reviews were included where acupuncture was compared with sham or placebo acupuncture, no treatment, or another intervention (pharmacological and non-pharmacological). We included Cochrane and non-Cochrane reviews and overviews of SRs. Systematic reviews containing non-RCT studies were included in order that information from RCTs could be extracted.

### 2.2. Exclusion Criteria

Reviews were excluded if they did not evaluate invasive acupuncture (e.g., reviews on acupressure or laser acupuncture). Systematic reviews were excluded if they evaluated acute pain but not chronic pain (e.g., specifically focusing on postoperative pain or pain in the emergency setting). Reviews focusing on additional elements such as bee venom were also excluded. Non-English reviews were included if they contained an English abstract. However, non-English reviews were not translated.

### 2.3. Evidence Synthesis

One review author (CAP) extracted information from reviews including type of pain, number of RCTs, treatments, conclusion and quality of evidence stated by the authors of each included review taken as a direct quote from the Conclusion, Abstract or Discussion sections of their manuscript. In addition, we ascribed a judgement of efficacy of each review according to whether the sample size met criteria based on the work of Moore et al. [16,17] and adopted by the Pain, Palliative and Supportive Care group from Cochrane Collaboration in their risk of bias assessment. They suggest that trial arms with fewer than 200 participants in RCTs or fewer than 500 participants in meta-analyses are at a high risk of bias, which seriously undermines confidence in findings. Thus, reviews were categorized as meeting our criteria for adequacy if they contained a pooled analysis of 500 events or at least one RCT with >200 participants in each arm of the trial. We categorised efficacy as: Sufficient evidence and in favour of acupuncture (+), sufficient evidence in favour of control/placebo (−), sufficient evidence but conflicting/inconclusive (=) and insufficient evidence to make a judgement (?). We also noted statements within manuscripts about RCT methodology across the following themes:

- The nature of placebo/sham interventions.
- Quality and risk of bias (including blinding).
- Sample size in relation to treatment effect. We used criteria developed by Dechartres [18] when commenting on adequacy of sample size as: adequately powered (≥200 patients per treatment arm), moderately powered (100–199 patients per treatment arm) and underpowered <100 patients per treatment arm).

## 3. Results

A total of 177 reviews of acupuncture for pain relief published between 1989 to September 2019 were included (Table 1). There were two overviews of Cochrane reviews, ten overviews of non-Cochrane SRs and 145 non-Cochrane SRs. The earliest systematic reviews were published in 1989 by ter Riet [19,20,21]. There were 20 Cochrane SRs (including updates), with the earliest published in 2000 by Tulder et al. [22] and the most recent published in 2018 by Choi et al. [23]. Findings are presented according to the most frequent evaluations of acupuncture for different types of pain and described chronologically to provide a sense of the evolution of evidence over time. A statement of current clinical guidance from NICE is provided where available.

### 3.1. Chronic Pain Irrespective of Aetiology or Pathophysiology

The earliest SR that evaluated the efficacy of acupuncture across chronic pain conditions irrespective of aetiology or pathophysiology was published in 2000 and was inconclusive, although it was claimed that six or more sessions of acupuncture were more likely to be associated with positive outcomes [24]. The first overview of SRs was published in 2006 and concluded that acupuncture was not shown to be efficacious for a variety of pain conditions [25].

We found four other overviews of SRs of acupuncture for chronic pain irrespective of aetiology or pathophysiology. In 2010, Ernst and Lee published an overview of 30 SRs of acupuncture (319 RCTs) for ‘rheumatic conditions’ and judged there to be some evidence to support efficacy in routine care of patients with pain associated with osteoarthritis, low back pain and lateral elbow pain [26]. Hopton et al. pooled data from eight meta-analyses of acupuncture for chronic pain and concluded that acupuncture was more effective than a placebo, despite an absence of statistical significance for individual conditions, except osteoarthritis of the knee and headache [27]. Two overviews published in 2011 concluded that there was tentative evidence that acupuncture might be effective for headache, peripheral joint osteoarthritis and neck pain (overview of eight Cochrane Reviews [28], overview of 57 SRs [29]), although reviewers agreed that the quality of the primary studies was poor, with a high risk of bias.

We found 20 SRs of acupuncture for chronic pain irrespective of aetiology or pathophysiology. In 2014, SRs reported that evidence supported the efficacy of wrist-ankle acupuncture and auricular acupuncture for alleviating chronic pain [30,31]. Since then, SRs were generally inconclusive because of methodological shortcomings and small sample sizes in primary studies [32,33,34,35,36]. In 2018, Vickers et al. concluded that evidence supported the efficacy of acupuncture for various chronic pain conditions associated with musculoskeletal disorders, headache and osteoarthritis, with beneficial effects persisting at long-term follow-up (39 RCTs, [37]). The long-term effects of acupuncture were consistent with evidence from an earlier SR by MacPherson et al. [38].

Evidence from SRs suggests that there are insufficient high-quality RCTs to judge the efficacy of acupuncture for chronic pain associated with various medical conditions. There is no specific NICE guidance about the use of acupuncture for chronic pain conditions irrespective of aetiology or pathophysiology, although some guidance exists for specific pain conditions (see respective sections below). Guidance by NICE on chronic pain assessment and management is currently being developed (GID-NG10069) with publication expected in August 2020.

### 3.2. Headache (Including Migraine)

We found one overview of Cochrane reviews of acupuncture for various pain conditions [28] (described above) that claimed there to be evidence that acupuncture was effective for tension-type headache (1 Cochrane review [45]) and migraine (1 Cochrane review [51]).

The earliest SR was published in 1999 and judged there to be too few RCTs of sufficient methodological quality to determine efficacy of acupuncture for recurrent headache (22 randomised or ‘quasi’ randomised trials, Melchart [54]) or tension-type and cervicogenic headache (8 RCTs [47]). A similar pattern of ‘promising’ but not definitive evidence continued through the next decade (27 RCTs [53]; 8 RCTs [46]), including a Cochrane review of 26 RCTs of acupuncture for idiopathic headache [52]. Nevertheless, some reviewers have claimed that there is evidence that acupuncture is superior to sham for chronic headache (31 RCTs, only 2 RCTs were of high quality and adequately powered, Sun [48]), and a recent Cochrane review providing evidence of superiority of acupuncture over placebo for the prevention of tension-type headache ([44] 12 RCTs, including two adequately powered RCTs) and episodic migraine ([49], 22 RCTs, including two adequately powered RCTs). A systematic review published in 2016 is consistent with the latter finding that acupuncture was superior to sham acupuncture for migraine (10 RCTs [50]).

Evidence from the SRs suggests that acupuncture prevents episodic or chronic tension-type headaches and episodic migraine, although long-term studies and studies comparing acupuncture with other treatment options are still required. The current NICE guidance (clinical guideline CG150) is that a course of up to 10 sessions of acupuncture over 5–8 weeks is recommended for tension-type headache and migraine [12].

### 3.3. Osteoarthritis (OA)

The overview of eight Cochrane reviews of acupuncture for various pain conditions described previously [28], judged there to be evidence that acupuncture produced short-term improvements in pain based on a SR of 16 RCTs on peripheral joint osteoarthritis [69]). In 2019, an overview of non-Cochrane SRs that included a meta-analysis concluded that acupuncture was beneficial for alleviating pain associated with OAK, although RCT outcomes assessed using the Grades of Recommendation, Assessment, Development and Evaluation (GRADE) indicated that the SR evidence was of mixed quality (12 SRs [55]).

We found that the earliest SR on acupuncture for OA was published in 1997 and found studies to be contradictory with no evidence that acupuncture was more effective than sham (9 RCTs [71]). SRs on acupuncture for peripheral joint OA were inconclusive in 2006 (18 RCTs [70]), superior to waiting list controls in 2010 ([69], Cochrane review of 16 RCTs) and associated with reductions in pain intensity, improvement in functional mobility and quality of life in 2014 (12 RCTs [68]). In 2018, a Cochrane review by Manheimer et al. found little evidence that acupuncture significantly reduced pain associated with OA of the hip [67]. To date, the majority of SRs have evaluated the clinical efficacy of acupuncture for OA of the knee (OAK).

We found that the earliest SR on acupuncture for OAK was published in 2001 and was inconclusive (7 RCTs, with 4 of low methodological quality [66]). Chronologically, SRs in 2002 (4 RCTs, [65]) and 2007 (11 RCTs [64]) were inconclusive, whereas a SR by Bjordal et al. in 2007 found statistically significant and clinically relevant short-term pain relief from two to four weeks of intensive electroacupuncture (7 RCTs [63]). In 2008 and 2012, two further SRs (10 RCTs [62]; 14 RCTs, [61] respectively) reported superiority over sham acupuncture. More recently, SRs in 2016 (10 RCTs [59]), 2017 (17 RCTs [72], 11 RCTs [58]), 2018 (16 RCTs [57]) and 2019 (8 RCTs [56]) judged there to be evidence that acupuncture provides relief of pain associated with OAK when administered alone or in combination with other treatments.

These positive findings are supported by a network meta-analysis published in 2013 that evaluated 22 treatments, including acupuncture (11 RCTs [60]), and judged there to be evidence of short-term efficacy. This finding was confirmed in another network meta-analysis published in 2018, which found that needle or electro-acupuncture decreased pain compared with other treatments (16 RCTs [57]). Nevertheless, reviewers consistently mitigate these positive findings by describing RCTs as having low methodological quality, thus reducing confidence in judgements.

The most recent evidence from a Cochrane review of 16 RCTs suggests that acupuncture is not superior to sham acupuncture for OA of the hip [67], although in contrast, evidence from non-Cochrane reviews suggests that there is moderate-quality evidence that acupuncture may be effective in the symptomatic relief of pain from OA of the knee. Why there should be a difference in evidence between the knee and the hip is not known. Interestingly, guidance from NICE (CG177) states: “Do not offer acupuncture for the management of osteoarthritis” Section 1.4.6. [198].

### 3.4. Chronic Low Back Pain and/or Neck Pain

The overview of eight Cochrane reviews of acupuncture for various pain conditions described previously [28] included one Cochrane review on low back pain [88] and judged there to be evidence that acupuncture might be an effective adjunctive intervention for low back pain. However, the quality of the primary studies was low. An overview of 16 SRs on acupuncture for low back pain published in 2015 judged that acupuncture either in isolation or as an adjunct to conventional treatment had short-term benefits but again, the quality of the included reviews was variable [78].

We found that the earliest SR on acupuncture chronic low back pain was published in 1989 and evaluated the clinical efficacy of acupuncture for neck and/or back pain (22 studies including 16 RCTs [19]) but the findings were inconclusive. The earliest SR that evaluated the clinical efficacy of acupuncture specifically for chronic low back pain was published in 1998 by Ernst et al. (12 RCTs [94]), and found evidence that acupuncture was superior to various control interventions, but insufficient evidence to judge whether it was superior to placebo. The included studies were mostly of high-quality, but sample sizes were inadequate. In 2000, Smith et al. published a SR that found no evidence that acupuncture was effective for either chronic neck or low back pain (13 RCTs [99]).

Throughout the following decade, SRs reported insufficient high-quality evidence to make any judgement on efficacy of acupuncture in treating low back pain (4 RCTs [93], 11 RCTs [22], 5 RCTs [91], 6 RCTs [95], 33 RCTs [89], 35 RCTs [88] and 10 RCTs [90]). In 2010, Trigkilidas published a SR that evaluated the clinical efficacy of acupuncture for low back pain (4 RCTs [85]) that judged there to be evidence of superiority of acupuncture compared with usual care in treating chronic low back pain, especially when patients have positive expectations about the intervention. Between 2011 and 2017, none of the published SRs provided compelling evidence to support the efficacy of acupuncture for chronic low back pain (2 RCTs [84], 7 RCTs [83], 13 RCTs [82], 32 RCTs [81], 8 RCTs [79] and 7 RCTs [77]), or lumbar spinal stenosis (12 studies, 6 RCTs [80]).

In 2018, Hu et al. published a SR that evaluated the clinical efficacy of acupuncture for low back pain (16 RCTs [75]) and found evidence that dry needling was more effective for low back pain than conventional acupuncture or sham immediately post treatment, but at follow-up, was equal to acupuncture. In 2018, Tang et al. published a SR that evaluated the clinical efficacy of acupuncture for the relief of pain associated with lumbar disc herniation (30 RCTs [76]), which found insufficient robust evidence to draw firm conclusions because of methodological shortcomings in primary RCTs. However, there was tentative evidence that dry needling was more beneficial than lumbar traction, drug therapy or Chinese herbal medicine. In 2019, Xiang et al. published a SR on acupuncture for non-specific low back pain (14 RCTs [74]). There was moderate evidence of benefit but confidence in the results was diminished due to heterogeneity and small sample sizes in the included studies.

Evidence suggests that there are insufficient high-quality RCTs to judge the efficacy of acupuncture for low back pain. In 2009, NICE published guidance for the management of non-specific low back pain that recommended a course of acupuncture as part of first line treatment [10]. This guidance produced much debate. Subsequently, NICE have updated guidance for the management of low back pain and sciatica in people over 16 (NG59) and currently recommend in Section 1.2.8 “Do not offer acupuncture for managing low back pain with or without sciatica”, even though the evidence had not significantly changed [9].

### 3.5. Myofascial Pain Syndrome and Myofascial Trigger Points

We found that the earliest SR on acupuncture (dry needling) to alleviate pain associated with myofascial trigger points (MTPs) was published in 2001 (23 RCTs [116]) and found no evidence to demonstrate the efficacy of any needling technique beyond placebo. In 2009, Tough et al. published a SR that evaluated the efficacy of dry needling acupuncture (7 RCTs [115]) which produced insufficient evidence to determine efficacy. A further systematic review by Tough et al. published in 2011 (3 RCTs [113]) had similar conclusions. In 2013, a SR found that acupuncture was superior to sham or placebo in reducing pain associated with upper quadrant myofascial pain immediately post-treatment and at four weeks, although the quality of the primary studies was low (12 RCTs [112]). In 2014, Ong and Claydon published a SR that evaluated the clinical efficacy of dry needling to alleviate pain associated with MTPs in the neck and shoulders (5 RCTs [111]) and found that there was no significant difference between dry needling and lidocaine.

In 2017, Espejo-Antúnez et al. published a SR that evaluated the clinical efficacy of dry needling to alleviate pain associated with myofascial trigger points (15 RCTs [106]) and found a possible short-term benefit following dry needling. In 2017, SRs have found tentative evidence that acupuncture alone or combined with other therapies improved outcomes associated with myofascial pain syndrome (10 RCTs [108]; 33 RCTs [107]), although substantial heterogeneity and a high risk of bias, including inadequate sample sizes in the primary RCTs, undermined confidence in the findings.

Evidence from SRs suggests that dry needling acupuncture might be effective in alleviating pain associated with myofascial trigger points, at least in the short-term, although there are insufficient high-quality RCTs to judge the efficacy with any degree of certainty. There is no guidance from NICE on the management of myofascial pain syndrome.

### 3.6. Cancer Pain

We found one overview of SRs of acupuncture for palliative and supportive cancer care that included 7 SRs [125], but only one systematic review on cancer-related pain [126]. We found that the earliest SR on acupuncture for pain associated with cancer and/or its treatment (7 studies with 3 RCTs [126]) concluded that there was evidence of efficacy for chemotherapy-induced nausea and vomiting, but insufficient evidence to judge efficacy for cancer-related pain. In 2010, a SR of 7 RCTs provided tentative evidence that that acupuncture alleviated cancer-related pain [124], and in 2011, the first Cochrane review on acupuncture for cancer pain judged there to be insufficient evidence to determine the efficacy (3 RCTs [123]), and this was confirmed in an update in 2015 (5 RCTs [119]). Subsequently, non-Cochrane SRs in 2012 (15 RCTs [122]), 2013 (11 RCT [121]), 2014 (33 studies, 6 RCTs [120]) and 2016 (20 RCTs [120]) provide promising but inconclusive evidence of efficacy. In 2017, Chiu et al. published a Cochrane review that evaluated the clinical efficacy of acupuncture for cancer-related pain, which included treatment-related or surgery-related pain, and judged there to be evidence that acupuncture alleviated pain associated with malignancy (29 RCTs [117]), but there was a high risk of bias due to inadequate sample sizes.

Evidence from the SRs suggests that there are insufficient high-quality RCTs to judge the efficacy of acupuncture for cancer-related pain and more high-quality, appropriately designed and adequately powered studies are needed. The most recent guidance from NICE (CSG4) recognises that patients who are receiving palliative care often seek complementary therapies, but it does not specifically recommend acupuncture. It recognises that “Many studies have a considerable number of methodological limitations, making it difficult to draw definitive conclusions” (Section 11.27) [199].

### 3.7. Fibromyalgia

We found that the earliest SR on acupuncture for fibromyalgia was published in 1999 (7 studies, 3 RCTs [136]) and concluded that there was limited evidence supporting the use of acupuncture for fibromyalgia but this was based on only one high-quality study. Subsequently, SRs published in 2007 (5 RCTs [135]) and 2009 (6 RCTs [134]) concluded that acupuncture had no symptomatic benefit, and in 2010 were inconclusive (7 RCTs [133], and 25 studies, 12 RCTs [132] respectively).

In 2013, a Cochrane review conducted by Deare et al. (9 RCTs [131]) found low-quality evidence that acupuncture might be superior to no acupuncture or medication, and moderate-quality evidence that acupuncture was not superior to sham. Non-Cochrane SRs published in 2013 (16 RCTs [130]) and 2014 (9 RCTs [129]) were inconclusive. In 2019, two SRs have produced evidence that acupuncture was superior to sham but the evidence status was downgraded due to high levels of heterogeneity and inadequate sample sizes (10 RCTs [128]; 12 RCT [127]).

Evidence from SRs suggests that there are insufficient high-quality RCTs to judge the efficacy of acupuncture for fibromyalgia pain. There is no NICE guidance on the treatment of fibromyalgia.

### 3.8. Pelvic Pain

We found one overview of SRs on acupuncture for primary dysmenorrhoea which was published in 2018 and concluded that the evidence was inconclusive (5 SRs [138]). We found a number of SRs on acupuncture and associated therapies for primary dysmenorrhea, although all report a high-risk of bias leading to evidence that is inconclusive (30 RCTs [147]; 4 RCTs [146]; 16 RCTs [144]; 20 RCTs [145]; 23 RCTs [142]; 60 RCTs [139]). In 2008, a SR investigating acupuncture for pelvic and back pain during pregnancy was inconclusive (3 RCTs [152]), and in 2010, a SR described RCTs findings as ‘promising’ but inconclusive for primary dysmenorrhea (27 RCTs [151]). A Cochrane review by Zhu et al. in 2011 on acupuncture for endometriosis included one low-quality RCT and was inconclusive [150]. A follow-up non-Cochrane review in 2017 including 10 RCTs was still inconclusive [143].

We found five SRs on acupuncture for chronic prostatitis and/or chronic pelvic pain, and despite promising RCT findings, all reviewers concluded that the evidence was inconclusive (9 RCTs [149]; 35 RCTs [148]; 7 RCTs [141]; 4 RCTs [140]; 4 RCTs [137]).

Evidence from the SRs suggests that there are insufficient high-quality RCTs to judge the efficacy of acupuncture for primary dysmenorrhea or chronic pelvic pain. There is NICE guidance on endometriosis (NG73) [200] but this does not recommend any form of Chinese medicine for this type of pelvic pain, although acupuncture is not specifically mentioned.

### 3.9. Inflammatory Arthritis

In 2018, an overview of SRs concluded that acupuncture has minimal or no impact on joint pain associated with rheumatoid arthritis (7 SRs, 20 RCTs [153]). We found that the earliest SR on acupuncture for pain associated with inflammatory rheumatic diseases was published in 1997 and found insufficient high-quality evidence to make a judgement on efficacy. Subsequently, a Cochrane review published in 2005 (2 RCTs [159]) and various non-Cochrane SRs published in 2008 (8 RCTs [158]; 8 RCTs [157]), 2013 (10 RCTs [156]) and 2016 (28 RCTs [155]) have been inconclusive. The most recent SR on acupuncture for rheumatoid arthritis reported that RCT findings were tentatively positive but inconclusive (13 RCTs [154]).

Evidence from the SRs suggests that there are insufficient high-quality RCTs to judge the efficacy of acupuncture for pain in inflammatory arthritis. There is a NICE guideline (NG100) [201] for the treatment of rheumatoid arthritis but this does not recommend acupuncture.

### 3.10. Neuropathic Pain/Neuralgia

The earliest SR on acupuncture for neurological symptoms was published in 1997 and reported that findings were positive for alleviation of symptoms associated with lumbar disk herniation (38 studies, 7 RCTs [171]).

The majority of SRs have been conducted on peripheral neuropathy of various aetiologies, but all had methodological shortcomings resulting in inconclusive evidence (chemotherapy-induced peripheral neuropathy, 6 studies, 3 RCTs [168]; various peripheral neuropathies, 15 studies, 13 RCTs [166]; and diabetic peripheral neuropathy, 14 RCTs [164]).

There are two SRs on acupuncture for trigeminal neuralgia (12 RCTs [170]; 33 RCTs [162]), two SRs on acupuncture for carpal tunnel syndrome (6 RCTs [169]; 12 RCTs [23]) and one SR on acupuncture for post-herpetic neuralgia (7 RCTs [165]). None were able to judge efficacy with any degree of confidence due to insufficient high-quality RCTs.

Evidence from the SRs suggests that there are insufficient high-quality RCTs to judge the efficacy of acupuncture for neuropathic pain or neuralgia. There is NICE guidance (CG173) [202] on the management of neuropathic pain, but acupuncture is not included in the list of recommended/not recommended treatments.

### 3.11. Other Pain Conditions

In 2002, a Cochrane review found insufficient high-quality RCTs to determine the efficacy of acupuncture for lateral elbow pain (4 RCTs [194]). In 2005, a Cochrane review found insufficient high-quality RCTs to judge the efficacy of acupuncture for shoulder pain. In 2011, a Cochrane review by Smith et al. found insufficient evidence to judge the efficacy of acupuncture or acupressure for labour pain (13 RCTs [187]).

Our search found an additional 27 reviews for a variety of other pain conditions, including dental/facial pain, osteoporosis and upper extremity pain of various aetiologies, although none of these reviews provides sufficient high-quality evidence to make a judgement about the efficacy of acupuncture (Table 1) [21,172,173,174,175,176,177,178,179,180,181,182,183,184,185,186,187,188,189,190,191,192,193,194,195,196,197].

Evidence from SRs suggests that there are insufficient high-quality RCTs to judge the efficacy of acupuncture for a variety of other painful conditions, including lateral elbow pain, shoulder pain and labour pain. There is no guidance available from NICE on the treatment of any of these conditions.

## 4. Discussion

Our evidence synthesis reveals long-standing and continued uncertainty about the clinical efficacy of acupuncture to alleviate pain, despite a high volume of published research. We have revealed a raft of SRs with inconclusive findings due to persistent methodological shortcomings in RCTs contributing to a high risk of bias and downgrading of evidence. These shortcomings include inadequate statistical power, uncertainty about adequacy of acupuncture technique and dose, and inappropriate design of ‘placebo’ acupuncture controls. These contribute to methodological and clinical heterogeneity, deterring systematic reviewers from pooling data for meta-analyses. When meta-analyses are conducted, substantial statistical heterogeneity results, markedly reducing confidence in findings and inferences [18,203,204]. The high financial cost of continuing to undertake research that produces inconclusive evidence is of concern and demands reconsideration of the methodological design and delivery of future RCT design. We will discuss three common challenges to the design of RCTs of acupuncture that emerge from our evidence synthesis: adequate sample sizes, adequate acupuncture intervention, and adequate placebo controls.

### 4.1. The Challenge of Inadequate Sample Sizes

RCTs with small sample sizes are associated with an overestimation of treatment effects. Dechartres et al. [18] found that treatment effects were, on average, 48% larger in trials with fewer than 50 patients. Overestimation of treatment effects occurs in studies with sample sizes of 100–200 participants per treatment arm, suggesting that at least 200 participants per treatment arm is necessary to achieve a low risk of bias. Roberts [205] argued that the production of fewer but broader reviews that exclude underpowered trials would increase the validity of review findings and create a more trustworthy evidence base. Turner et al. [203] examined the distribution of statistical power within meta-analyses published as part of Cochrane reviews and argued that the results of meta-analyses that contain at least two adequately powered studies are not influenced to any significant degree when underpowered studies are omitted. At present, the inclusion of underpowered studies in meta-analyses is at the discretion of reviewers.

Funding constraints that prevent the use of larger sample sizes in RCTs is likely to continue into the future. Thus, strategies to reduce statistical heterogeneity associated with high variance in pain data in RCTs need consideration. Often, pain data used as the primary outcome within RCTs is a continuous variable, such as pain intensity measured on a visual analogue scale (VAS) and expressed as an average. Averages of pain intensity data from VAS can be misleading because averages may obscure good and poor responders to acupuncture [206,207]. There is a likelihood that scores of pain intensity produce U-shaped rather than bell-shaped distributions, with some participants experiencing large reductions in pain and others not. Thus, pain intensity data from acupuncture responders may be diluted by data from non-responders [208]. For this reason, the Pain and Palliative Support and Care group of the Cochrane collaboration recommends the use of primary outcome responder rates of participants reporting relief of 30% or greater (i.e., at least moderate pain relief) or 50% or greater (i.e., significant pain relief) expressed as frequency (dichotomous) data.

### 4.2. The Challenge of Appropriate Controls

Acupuncture RCTs can assess two aspects of the active ingredient of treatment: effects associated with needling acupuncture points and effects associated with needles piercing the skin. Thus, two common controls used in RCTs of acupuncture are: inserting real needles into the skin at non-acupuncture points and using ‘sham’ needles which touch but do not penetrate the skin. It is important that SRs and RCTs emphasise exactly which outcome is being assessed at the outset, and ideally include this in the title and aim of the report.

Controls that involve inserting needles into the skin at non-acupuncture points can be used to determine the influence of needling discrete points of the skin on outcome. If administering treatment at any point on the skin produced equivalent benefits and harms when compared with needling specific points, this would challenge the need for anatomical acupuncture charts and prescribed acupuncture practitioner training.

Controls that use ‘sham’ needles which touch but don’t penetrate the skin are often labelled as placebo controls. The purpose of a placebo control comparison is to isolate the effect of the act of receiving a treatment from the active ingredient of the treatment. Placebo controls are usually operationalised using fake or sham interventions and enable measurement of non-specific treatment effects associated with expectations, conditioning, anxiety and social context (i.e., therapist/patient interaction and theatrical elements of the treatment) [209,210]. It has been argued that the reason why some RCTs fail to detect differences in treatment effects between real and sham acupuncture is that sham needling techniques are not physiologically inert, and this may have contributed to an underestimation of acupuncture effects in the evidence base [211]. This argument is valid but can be misleading if taken at face value. The purpose of a control intervention is not to be physiologically inert but rather to control for outcomes associated with non-specific effects of the act (theatre) of receiving the treatment. No placebo control (including a sham needling) is ever physiologically inert because it instigates changes in physiological (and psychological) state. The human body evolved to detect and respond to disturbances in the internal and/or external environment (i.e., stimuli) from physical, physiological, social and/or environmental change. This is the premise of homeostasis.

Placebo controls are research tools that enable isolation of effects associated with the active ingredient(s) of the treatment. Thus, a comparison of effects during real needling versus sham needling, whereby needles touch but do not penetrate the skin, enable investigators to isolate the magnitude and incidence of effects associated with needles piercing the skin per se (i.e., the ‘acu’ and ‘puncture’). If puncturing the skin with needles produces equivalent benefits to touching without puncturing the skin, then it may be safer not to puncture the skin in clinical practice, providing that the sham needles do less harm. Interestingly, a system of evaluating the physiological effects of sham needling has been proposed to assist researchers [212].

The term ‘placebo’ is used extensively in research and clinical literature, although it lacks scientific precision and has become emotive. We would prefer precise statements of purpose and method when describing control interventions. For example, a control group that uses fake needles that do not puncture the skin would be used to isolate effects associated with needles puncturing the skin. We would also encourage a shift away from assessing patient ‘blinding’ using questions such as ‘Do you think the intervention was a placebo?’ to questions assessing the ‘credibility’ and ‘functioning’ of interventions using questions such as ‘Do you think the intervention was credible?’ and ‘Do you think the intervention was functioning correctly?’, as has been suggested for other non-pharmacological interventions such as transcutaneous electrical nerve stimulation (TENS) [213].

### 4.3. The Challenge of Adequacy of Dose

Acupuncture practitioners argue that acupuncture is a complex intervention that should not be standardised but instead tailored to each individual patient, based on principles of practice and the experience of the clinician. Components of needling include type, number, and location of needles, needling technique (e.g., thrusting, rotation, flicking, pecking), duration of needle insertion, regimen of treatment and philosophical paradigm. Debates about optimal technique are long-standing and there are evidence-based principles underpinning optimisation of technique for acupuncture treatment [214]. Delivering identical acupuncture prescription to all participants runs the risk of some participants receiving sub-optimal dose. Often, acupuncture interventions used in RCTs are grounded in principles of Western acupuncture with flexibility to individualise treatment at the discretion of individual practitioners. Individualising acupuncture treatment increases between-subject variability in treatment (e.g., needling number, location, technique, duration). At face value, this may appear to conflict with classical RCT methodology that aims to standardise methodology and treatment intervention under strictly controlled conditions. However, standardisation can be based on the principles of optimising treatment per individual, as is the case when titrating drug dosage to therapeutic window. What constitutes adequacy of acupuncture technique and dose has been a matter of much debate [56,214,215,216].

In trials of pharmacological agents, dose is crucial, and it should be no different in studies investigating the efficacy of acupuncture. The Standards for Reporting Interventions in Controlled Trials of Acupuncture (STRICTA) were developed from the consolidated standards for reporting trials (CONSORT) [217] to encourage accurate reporting of the acupuncture intervention [218]. STRICTA recommend that six items should be included: rationale, details of needling (e.g., points used, depth, angle, needle thickness, number of needles), treatment regimen, co-intervention, practitioner background and control interventions [219]. The impact of using STRICTA has been positive with improvements in reporting quality of RCTs on acupuncture [220,221,222,223]. In 2008, White et al. published a meta-analysis that provided evidence that better outcomes in comparisons of acupuncture with non-acupuncture controls were achieved when noted that greater numbers of needles and treatment sessions were used [214]. In 2019, Sun et al. conducted a systematic review of eight RCTs (2106 participants) to determine whether the effect of acupuncture is dose-dependent for symptom management in knee osteoarthritis [56]. Sun et al. proposed a scoring system whereby +1 score was awarded if ≥9 points needled, if de qi was present, if ≥2 treatment sessions a week and if ≥8 treatment sessions in total. A score of −1 was awarded to each of these parameters if they were below these thresholds. The sum of scores was taken and high dosage categorised for total between 1 and 4, medium dosage for a score of 0 and low dosage for scores from −4 to −1. Sun et al. categorised one RCT as low dose, one RCT as medium dose and 6 RCTs as high dose and concluded that higher dosage of acupuncture was associated with better pain relief and functional improvements. It is becoming common for journal editors to require STRICTA in RCTs of acupuncture and this will improve comparison and assessment of adequacy of acupuncture dose in systematic reviews. What is less common, however, is the inclusion and reporting of ‘run-in phases’ in RCTs, whereby optimisation of technique and dosage is titrated over a period of weeks prior to randomisation into real and placebo acupuncture.

### 4.4. Design of Future Randomised Controlled Trials (RCTS)

It has been argued that enriched enrolment with randomised withdrawal (EERW) study designs are of value for treatments influencing symptoms but not necessarily the course of the underlying disease or pathology, as is the case for acupuncture in the management of chronic pain [224]. The potential for using such designs in the assessment of pharmacological agents has been recognised [225], although EERW designs are rarely used to assess non-pharmacological interventions. The EERW trials consist of (i) an observational ‘open-label’ phase with all participants receiving active treatment (acupuncture), during which treatment technique and dosage would be titrated and optimized, followed by (ii) a RCT phase, whereby participants who had potential for response were enrolled (i.e., an enriched sample) and randomised to receive either experimental (real needling) or control interventions (sham needling). Selection of participants for the enriched sample of the RCT is based on the findings from phase one and would exclude participants who did not wish to continue treatment or experienced non-manageable adverse events, although their data from phase one would be analysed. Trials with EERW designs increase sensitivity to detect treatment effects by enriching the sample of participants enrolling into the randomised controlled phase of the trial, thus reducing the need for large sample sizes [207].

To our knowledge, there have not been any published studies of acupuncture using the EERW design, although it has been used to determine the efficacy of drugs for chronic pain conditions. Given the shortcomings in classically design RCTs on acupuncture, it would be interesting to observe the results of studies using an EERW design.

### 4.5. Limitations of This Review

A limitation of this synthesis is that it does not contain granular quantitative analyses. It could be argued that there is a case for an all-encompassing SR and meta-analysis of all RCTs on acupuncture for pain conditions, but this would be a considerable undertaking with the possibility that it not produce any meaningful information due to the relatively poor quality of RCTs resulting in amplification of heterogeneity.

## 5. Conclusions

We hope that our evidence synthesis of systematic reviews and meta-analyses of RCTs of acupuncture for chronic pain conditions serves as a reference tool for practitioners, researchers and commissioners. Our evidence synthesis reveals a long-standing unresolved debate about the clinical efficacy of acupuncture to alleviate pain that is grounded in a high volume of inconclusive RCT evidence. If healthcare providers and commissioners are to be able to make informed choices on the role of acupuncture for chronic pain, it is essential that the quality of clinical trials of acupuncture is improved. Our evidence synthesis has revealed three methodological challenges that have faced investigators of RCT of acupuncture for decades. We have argued that enriched enrolment with randomised withdrawal trial designs may provide a way forward. We hope that our review catalyses further debate on this issue.

## Figures and Tables

**Table 1:** Systematic reviews of Acupuncture (acup) for Chronic Pain Conditions.

Condition	Reference	Type of Review	Treatments Evaluated	No of RCTs in Review	Systematic Reviewers’ Conclusion of Efficacy *	Our Judgement of Efficacy **	Systematic Reviewers’ Conclusion of Quality of Available Evidence ***	Our Comments
**CHRONIC PAIN IRRESPECTIVE OF AETIOLOGY OR PATHOPHYSIOLOGY**								
	Vickers et al., 2018 [37]	Non-Cochrane Systematic Review	Chronic Pain	39	‘We conclude that acupuncture is effective for the treatment of chronic pain, with treatment effects persisting over time.’	+	‘… in keeping with the original analyses, significant heterogeneity was found in 5 out of 7 comparisons.’	Large studies with arms >200 participants were included for headache, low back pain, OA and shoulder pain.
	MacPherson et al., 2017 [38]	Non-Cochrane Systematic Review	Chronic Pain (persistence of acupuncture effects over time)	29	‘The effects of a course of acupuncture treatment for patients with chronic pain do not appear to decrease importantly over 12 months.’	+	‘… strict inclusion criteria required evidence of unambiguous allocation concealment, leading to our inclusion of only higher quality trials.’	Dataset of almost 18,000 patients, including some high-quality studies with >200 participants per trial arm.
	Gattie et al., 2017 [36]	Non-Cochrane Systematic Review	Musculoskeletal conditions	13	‘… evidence suggests that dry needling is more effective than no treatment, sham dry needling, and other treatments …’	?	‘… overall quality of the evidence was considered to be very low to moderate using the GRADE approach.’	Included studies all had arms of <200 participants.
	Zhang et al., 2017 [39]	Non-Cochrane Systematic Review	Pain conditions	23	‘Cupping therapy and acupuncture are potentially safe, and they have similar effectiveness in relieving pain.’	N/A	‘… no study was evaluated as low risk of bias, studies unclear risk of bias, and the remaining 15 studies, high risk of bias.’	This was a comparative SR between acupuncture and cupping. None of included studies had arms of >200 participants
	Cox et al., 2016 [32]	Non-Cochrane Systematic Review	Musculoskeletal Disorders of the Extremities	15	‘Evidence for the effectiveness of acupuncture for musculoskeletal disorders of the extremities was inconsistent.’	=	‘Ten of 15 RCTs had a low risk of bias …. Five of 15 RCTs had a high risk of bias.’	Effect sizes were small. One large study with >200 participants per treatment arm and low risk of bias.
	Yuan et al., 2016 [33]	Non-Cochrane Systematic Review	Musculoskeletal pain	61	‘Our review provided low-quality evidence that acupuncture has a moderate effect (approximately a 12-point pain reduction on the VAS 100 mm) on relieving pain associated with musculoskeletal disorders.’	=	‘The main weakness of this study was the relative paucity of high-quality RCTs. About half of the trials did not perform intention to treat analyses or correct allocation concealments.’	This review included several large studies with pooled events of >500
	Wong et al., 2015 [34]	Non-Cochrane Systematic Review	Chronic MSK pain	19	‘This review showed moderate evidence of local or distant points stimulation in reducing pain at the end of the treatment when compared with control groups.’	?	‘The 19 studies were of moderate quality.’	Comparison between local and distal acup stimulation. No included studies had arms of >200 participants.
	Zhao et al., 2015 [35]	Non-Cochrane Systematic Review	Chronic pain	15	‘Due to the significant clinical heterogeneity and methodological flaws identified in the analysed trials, the current evidence on AT for chronic pain management is still limited.’	?	‘The significant methodological flaws identified … contributed to high risk of bias of the included studies.’	Auricular therapy studies (not all acup). No included studies had arms of >200 participants.
	Yeh et al., 2014 [31]	Non-Cochrane Systematic Review	Pain management	22	‘… AA (auricular acup), was found to be a significant method of pain relief when compared to the sham or control group.’	?	‘In the studies included in this meta-analysis, 91% were rated as good [quality]…’	No included studies had arms of >200 participants. Publication bias was detected.
	Zhu et al., 2014 [30]	Non-Cochrane Systematic Review	Pain symptoms (Wrist-ankle acup (WAA))	33	‘… the efficacy of WAA or WAA adjuvants was much better than Western medicine, sham acupuncture, or body acupuncture.’	?	‘… higher quality and more rigorously designed clinical trials with large enough sample sizes are needed …’	All studies were Chinese. No included studies had arms of >200 participants.
	Vickers et al., 2012 [40]	Non-Cochrane Systematic Review	Chronic pain	29	‘Acupuncture is effective for the treatment of chronic pain and is therefore a reasonable referral option. Significant differences between true and sham acupuncture indicate that acupuncture is more than a placebo.’	+	‘Neither study quality nor sample size appear to be a problem for this meta-analysis, on the grounds that only high-quality studies were eligible, and the total sample size is large.’	Authors looked at musculoskeletal (MSK) pain, osteoarthritis (OA), headache and shoulder pain. Six studies included with arms of >200 participants.
	Ernst & Lee 2011 [29]	Systematic review of systematic reviews	Multiple pain conditions	57 SR	‘In conclusion, numerous systematic reviews have generated little truly convincing evidence that acupuncture is effective in reducing pain. ‘	−	‘For indications where only one systematic review was available, definitive conclusions were usually prevented by the paucity or poor quality of the primary studies or the poor quality of the reviews.’	Four out of 57 reviews were of excellent quality. Primary studies variable in sample sizes.
	Lee & Ernst 2011 [28]	Overview of Cochrane reviews	Pain	8 SR	‘All of these reviews were of high quality. Their results suggest that acupuncture is effective for some but not all types of pain.’	?	‘Many primary studies that were included … had a high risk of bias. This often means that the current evidence is limited, insufficient, or inconclusive.’	All these Cochrane Reviews are of high quality. Acupuncture effective for only some types of pain.
	Asher et al., 2010 [41]	Non-Cochrane Systematic Review	8 perioperative, 4 acute, and 5 chronic pain	17	‘Auriculotherapy may be effective for the treatment of a variety of types of pain, especially postoperative pain.’	?	‘… we believe our results likely reflect the results of higher quality studies and reduced publication bias.’	Auricular therapy only. Six studies were rated as ‘good’ quality. Study arms all had <200 participants.
	Ernst & Lee 2010 [26]	Overview of systematic reviews	Rheumatic conditions	30 SR	‘Only for OA, low back pain and lateral elbow pain is the evidence sufficiently sound to warrant positive recommendations of this therapy …’	=	‘SRs of acupuncture have been noted to be limited by the often poor-quality of the primary data …’	Studies of variable quality and primary studies of various sample sizes.
	Hopton & MacPherson 2010 [27]	Systematic review of pooled data from meta-analyses	Chronic Pain	8 SR	‘The accumulating evidence from recent reviews suggests that acupuncture is more than a placebo ….’	=	‘… the reviews we are reporting include small-scale trials, with some variability in quality …’	Positive score for OA knee and headache only. Number of pooled participants >1000 in 3 of SRs.
	Madsen et al., 2009 [42]	Non-Cochrane Systematic Review	Pain conditions	13	‘We found a small analgesic effect of acupuncture that seems to lack clinical relevance and cannot be clearly distinguished from bias.’	−	‘The review is fairly large, includes several trials of high methodological quality …’	One study with arms >200 participants and pooled events of >500.
	Ernst et al., 2009 [43]	Systematic review of Cochrane reviews	Multiple conditions, including pain.	32 SR	‘It is concluded that Cochrane reviews of acupuncture do not suggest that this treatment is effective for a wide range of conditions.’	−	‘…. acupuncture trials are … often poorly designed and badly reported.	Included 10 SRs on chronic pain conditions, representing 95 primary RCTs.
	Derry et al., 2006 [25]	Systematic review of systematic reviews 1996-2005	Multiple pain conditions	35 SR	‘Systematic reviews… provide no robust evidence that acupuncture works for any indication.’	−	‘Many reviews included studies with designs known to be associated with bias and overestimation of treatment effects.’	Included SRs on non-pain conditions, e.g., nausea and vomiting. 24 out of 35 reviews had information on less than 1000 patients.
	Ezzo et al., 2000 [24]	Non-Cochrane Systematic Review	Chronic pain	51	‘There is limited evidence that acupuncture is more effective than no treatment for chronic pain, and inconclusive evidence that acupuncture is more effective than placebo, sham acupuncture or standard care.’	?	‘Two-thirds of the studies … received a low-quality score and low-quality trials were significantly associated with positive results … High-quality studies were associated with … risk of false negative (type II) errors …’	Fifty-one RCTs representing 2423 chronic pain patients. The median sample size per group was 18 and the mode was 15.
**HEADACHE**								
**(a) Tension-type**								
	Linde et al., 2016 [44]	Cochrane Review	Episodic or chronic tension-type headache	12	‘… acupuncture is effective for treating frequent episodic or chronic tension-type headaches …’	+	‘Overall, the quality of the evidence assessed using GRADE was moderate or low ….’	Includes 2 studies with >200 participants in each study arm
	Linde et al., 2009 [45]	Cochrane review	Episodic or chronic tension-type headache	11	‘… acupuncture could be a valuable non-pharmacological tool in patients with frequent episodic or chronic tension-type headaches.’	+	‘… sequence generation, allocation concealment, handling of dropouts and withdrawals and reporting of findings were adequate.’	Includes 2 studies with >200 participants in each study arm
	Davis et al., 2008 [46]	Non-Cochrane Systematic Review	Non-migrainous headache	8	‘… limited efficacy for the reduction of headache frequency’	−	‘… all included studies to be of high quality, with scores of 3 or 4…’	One study with >200 participants per arm. Pooled analysis not significant
	Vernon et al., 1999 [47]	Non-Cochrane Systematic Review	Tension-type and cervicogenic headache	8	‘Acupuncture does not appear to be more effective than a course of physiotherapy.’	?	‘Two of four higher quality studies reported negative results ….’	None of included studies has >200 participants in each arm
	Sun et al., 2008 [48]	Non-Cochrane Systematic Review	Chronic headache	31	‘… acupuncture is superior to sham acupuncture and medication therapy in improving headache intensity, frequency, and response rate.’	+	‘The quality of the more recent trials is higher than the older trials, with more emphasis on proper randomization, allocation concealment, and description of patient dropout.’	Three studies with >200 participants in each arm. Pooled events >500
	Ter Riet et al., 1989 [20]	Non-Cochrane Systematic Review	Tension-type headache and migraine	10	‘It is not … possible to draw a conclusion that acupuncture works for migraine and/or tension headache’.	?	‘… number of patients and the methodological level of the experiments are … low.’	None of included studies has >200 participants in each arm
**(b) Migraine**								
	Linde et al., 2016 [49]	Cochrane review	Episodic migraine	22	‘… a course of acupuncture consisting of at least six treatment sessions can be a valuable option ….’	+	‘Overall the quality of the evidence was moderate.’	Number of pooled events >500 and 3 studies with >200 participants in each arm
	Yang et al., 2016 [50]	Non-Cochrane Systematic Review	Migraine	10	‘… verum acupuncture is superior to sham acupuncture in migraine’	?	‘The majority of the included studies were considered to be of generally high methodological quality ….’	All study arms <200 participants and pooled events <500
	Linde et al., 2009 [51]	Cochrane review	Migraine	22	‘… acupuncture is at least as effective as, or possibly more effective than, prophylactic drug treatment, and has fewer adverse effects.’	+	‘Methods for sequence generation, allocation concealment, handling of dropouts and withdrawals and reporting of findings were adequate in most of the recent trials.’	Number of pooled events >500 and 3 studies with >200 participants in each arm
**(c) Other headache**								
	Melchart et al., 2001 [52]	Cochrane review	Idiopathic headache	26	‘… the existing evidence supports the value of acupuncture for the treatment of idiopathic headaches.’	?	‘… the quality and amount of evidence are not fully convincing.’	None of included studies has >200 participants in each arm. Pooled events <500
	Manias et al., 2000 [53]	Non-Cochrane Systematic Review	Primary headaches	27	‘In the majority of the trials (23 of the 27 trials), it was concluded that acupuncture offers benefits in the treatment of headaches.’	?	The authors did not make a statement of study validity.	Insufficient information available regarding sample sizes.
	Melchart et al., 1999 [54]	Non-Cochrane Systematic Review	Recurrent headache	22	‘… no straightforward recommendation for clinical practice can be made. ‘	?	‘… most trials were small and were either inadequately reported or had identifiable methodological flaws.’	None of included studies has >200 participants in each arm. Pooled events < 500
**OSTEOARTHRITIS (OA)**								
**(a) Knee**								
	Li et al., 2019 [55]	Overview of Systematic Reviews	OA Knee	12 SRs	‘According to the high-quality evidence, we concluded that acupuncture may have some advantages in treating KOA.’	+	‘… there are some risk of bias and reporting deficiencies still needed to be improved.’	Two of the largest SRs were deemed to have the highest reporting quality.
	Sun et al., 2019 [56]	Non-Cochrane Systematic Review	Symptom management in OA knee	8	‘The effect of acupuncture may be associated with dose of acupuncture, with a higher dosage related to better treatment outcomes …’	+	‘The results of this study rely largely on high-quality primary RCTs. However, they are inevitably limited by the small number of included trials …’	One included study with >200 participants and one with 189–191 per trial arm.
	Li et al., 2018 [57]	Non-Cochrane Systematic Review	Symptom management in OA knee	16	‘… acupuncture with heat pain or electrical stimulation might be suggested as the better choices ……’	+	‘The methodological quality evaluation was low …’	Network meta-analysis. One study with >200 participants and two with 189–191 per sample arm.
	Chen et al., 2017 [58]	Non-Cochrane Systematic Review	Knee OA (KOA)—Electro acupuncture (EA) studies only	11	‘… EA is a great opportunity to remarkably alleviate the pain …’	?	‘… more high quality RCTs with rigorous methods of design, measurement and evaluation are needed.’	Meta-analysis with <500 pooled events.
	Lin et al., 2016 [59]	Non-Cochrane Systematic Review	OA knee	10	‘… only short-term pain relief in patients with chronic knee pain due to osteoarthritis.’	?	‘Significant publication bias was not detected (*p* > 0.05), but the heterogeneity of the studies was substantial.’	Insufficient information available on sample sizes of primary studies.
	Corbett et al., 2013 [60]	Non-Cochrane Systematic Review	OA knee	11	‘… acupuncture can be considered as one of the more effective physical treatments for alleviating osteoarthritis knee pain in the short-term.’	+	‘Around three-quarters of the studies were classed as being of poor quality.’	Network meta-analysis (2794 acup patients). Eleven “better-quality” acupuncture studies included.
	Cao et al., 2012 [61]	Non-Cochrane Systematic Review	OA knee	14	‘Acupuncture provided significantly better relief from knee osteoarthritis pain and a larger improvement in function than sham acupuncture, standard care treatment, or waiting for further treatment.’	+	‘According to the Cochrane Back Review Group scale, 11 RCTs had high internal validity and 3 RCTs had low internal validity.’	One study with >200 participants and four with >100 per trial arm. Pooled events > 500.
	Selfe et al., 2008 [62]	Non-Cochrane Systematic Review	OA knee	10	‘… acupuncture is an effective treatment for pain and physical dysfunction associated with osteoarthritis of the knee.’	+	Authors did not make any assessment of quality.	Included 1 study with >200 participants per trial arm and 2 with >100.
	Bjordal et al., 2007 [63]	Non-Cochrane Systematic Review	OA knee	7	‘… an intensive regimen of 2–4 weeks with TENS, EA or low-level laser therapy (LLLT) seems to safely induce statistically significant and clinically relevant short-term pain relief.’	?	‘Trials were generally of medium to high quality (≥3)…’ (Jadad)	Insufficient pooled events in EA studies (<500) which reduced validity of conclusions.
	Manheimer et al., 2007 [64]	Non-Cochrane Systematic Review	OA knee	11	‘Waiting list-controlled trials suggest clinically relevant benefits, some of which may be due to placebo or expectation effects.’	?	‘Because of heterogeneity and small effects, current estimates should be regarded as preliminary.’	One study with >200 participants and three with >100 per trial arm. No information on number of pooled events.
	Ferrández Infante et al., 2002 [65]	Non-Cochrane Systematic Review	OA knee	4	‘… not enough evidence to recommend acupuncture as a treatment for knee pain.’	−	‘Only one study presented a high-quality level …’	None of the included studies had sample sizes of >100
	Ezzo et al., 2001 [66]	Non-Cochrane Systematic Review	OA knee	7	‘The existing evidence suggests that acupuncture may play a role in the treatment of knee OA.’	?	‘More than half of the trials (n = 4) received a low-quality rating.’	None of included studies had trial arms of >200 participants
**(b) Hip**								
	Manheimer et al., 2018 [67]	Cochrane review	Hip OA	16	‘Acupuncture probably has little or no effect in reducing pain or improving function relative to sham acupuncture in people with hip osteoarthritis.’	−	‘Overall the evidence was limited, with only six RCTs of five different comparisons, with small sample sizes, and at high risk of bias, especially for the criteria of blinding.’	None of included studies had trial arms of <200 participants. Pooled events were <500.
**(c) Other**								
	Manyanga et al., 2014 [68]	Non-Cochrane Systematic Review	OA—Various	12	‘The use of acupuncture is associated with significant reductions in pain intensity, improvement in functional mobility and quality of life.’	+	‘… limited by methodological challenges... From the included trials, 75% were adjudicated to be of unclear or high risk of bias.’	One study >200 participants. Pooled events > 500. However, effect estimates might be inflated due to risk of bias in some studies.
	Manheimer et al., 2010 [69]	Cochrane review	Peripheral joint OA	16	‘Waiting list-controlled trials … suggest statistically significant and clinically relevant benefits … which may be due to expectation or placebo effects.’	+	‘… we considered the five [studies] with the highest quality ratings on the Cochrane Back Review Group scale. Only two of the five had any obvious methodological flaws …’	Pooled events were >500
	Kwon et al., 2006 [70]	Non-Cochrane Systematic Review	Peripheral joint OA	18	‘…. acupuncture seems an option worthy of consideration particularly for knee OA.’	=	‘Even though the total number of 18 RCTs is encouraging, it is too small considering the heterogeneity of the overall dataset.’	14 studies on OA knee. One large study including >200 participants in trial arms. Pooled events < 500.
	Ernst 1997 [71]	Non-Cochrane Systematic Review	OA	9 (13 studies)	‘… the notion that acupuncture is superior to sham-needling in pain associated with OA is not supported by published data from controlled clinical trials.’	−	‘Most trials suffer from methodological flaws.’	Sample sizes for all studies < 100 participants, therefore insufficient.
**CHRONIC KNEE PAIN (NON-SPECIFIC)**								
	Zhang et al., 2017 [72]	Non-Cochrane Systematic Review	Chronic knee pain	17	‘… we are currently unable to draw any strong conclusions regarding the effectiveness and safety of acupuncture for chronic knee pain.’	−	‘… the overall methodological quality of the included trials was not satisfactory.’	One study with trial arms of >200 participants and one with arms of 190/189.
	White et al., 2007 [73]	Non-Cochrane Systematic Review	Chronic knee pain	13	‘Acupuncture that meets criteria for adequate treatment is significantly superior to sham acupuncture and to no additional intervention …’	+	‘The evidence appears to be robust enough to encourage wider use of acupuncture for chronic knee pain …’	Included 3 large studies of high quality. Two with trial arms >200 participants and one with >189 participants.
**LOW BACK PAIN**								
**(a) Chronic**								
	Xiang et al., 2019 [74]	Non-Cochrane Systematic Review	Non-specific low back pain	14	‘… there is moderate evidence of efficacy for acupuncture in terms of pain reduction immediately after treatment … when compared to sham or placebo acupuncture’.	=	‘… trials included were heterogeneous regarding the needling sites, the needling manipulation and the duration of acupuncture sessions, and the type of sham ….’	One study with trial arms of >200 participants.
	Hu et al., 2018 [75]	Non-Cochrane Systematic Review	Chronic LBP	16	‘… current evidence is not robust to draw a firm conclusion regarding the efficacy and safety of DN for LBP.’	−	‘… methodological shortcomings … greatly reduced the quality of evidence.’	No studies with trial arms of >200. Pooled events < 500.
	Tang et al., 2018 [76]	Non-Cochrane Systematic Review	Lumbar disc herniation	30	There is tentative evidence that acupuncture is more beneficial at alleviating pain than lumbar traction, drug therapy or Chinese herbal medicine.	=	There was insufficient robust evidence to draw firm conclusions because of methodological shortcomings.	Pooled events > 500 participants. GRADE evidence assessed by authors was LOW or VERY LOW for all studies.
	Yeganeh et al., 2017 [77]	Non-Cochrane Systematic Review	Low back pain (in Iran)	3 (7 in total)	‘In conclusion, overall, the lack of studies with a low risk of bias precludes any strong recommendations.’	?	‘The methodological quality of the studies was generally poor.’	None of the 3 acup studies had arms of >200 participants and pooled events insufficient (<500)
	Liu et al., 2015 [78]	Overview of systematic reviews	Chronic low back pain	16 SR	‘…. consistent evidence shows that acupuncture is more effective for pain relief and functional improvement at short-term follow-ups.’	+	‘… three systematic reviews were considered as high quality, eight as moderate quality, and five as low quality …’	Number of pooled participants in moderate to high quality SRs were >500
	Close et al., 2014 [79]	Non-Cochrane Systematic Review	Low back and pelvic pain in pregnancy	6 (8 in total)	‘At present, we simply do not have enough high-quality trials on CAM for managing Low back and pelvic pain in pregnancy.’	−	‘The restricted availability of high-quality studies, combined with the very low evidence strength, makes it impossible to make evidence-based recommendations ….’	Overall strength of evidence graded VERY LOW. Study arms had sample sizes of <200.
	Kim et al., 2013 [80]	Non-Cochrane Systematic Review	Lumbar spinal stenosis	12 (6 RCT)	‘We found no conclusive evidence of the effectiveness and safety of acupuncture ….’	?	‘The current evidence found in this review is seriously limited by high or uncertain risk of bias.’	All studies had arms with <200 participants
	Lam et al., 2013 [81]	Non-Cochrane Systematic Review	Chronic, non-specific low back pain	32	‘…. acupuncture is effective in providing long-term relief of chronic low back pain, but this effect is produced by non-specific effects that arise from skin manipulation.’	=	‘Given the clinical heterogeneity of other treatments for chronic low back pain, it is not surprising that a consistent conclusion could not be made ….’	Two studies had trial arms with >200 participants, however, the results were not conclusive.
	Xu et al., 2013 [82]	Non-Cochrane Systematic Review	Chronic low back pain	13	‘Compared with no treatment, acupuncture achieved better outcomes in terms of pain relief, disability recovery and better quality of life, but these effects were not observed when compared to sham acupuncture...’	=	No specific statement on quality included. The authors state ‘The main biases that affected the results were performance bias and detection bias.’	Two studies had trial arms with >200 participants, however, the results were not conclusive.
	Hutchinson et al., 2012 [83]	Non-Cochrane Systematic Review	Chronic, non-specific low back pain	7	‘This review provides some evidence to support acupuncture as more effective than no treatment …’	−	No specific statement on quality of studies.	3 studies with trial arms of >200 participants but these studies did not demonstrate a significant difference between acupuncture and sham.
	Standaert, et al., 2011 [84]	Non-Cochrane Systematic Review	Chronic low back pain	2	‘… insufficient evidence to comment on the relative benefit of acupuncture compared with either structured exercise or SMT…’	?	‘The overall strength of the evidence … was “insufficient.”	Results of only one acup RCT included therefore there was insufficient evidence.
	Trigkilidas 2010 [85]	Non-Cochrane Systematic Review	Chronic low back pain	4	‘acupuncture can be superior to usual care in treating chronic low back pain, especially, when patients have positive expectations about acupuncture.’	=	No specific statement on quality was made but study designs introduced bias.	3 out of 4 studies had trial arms of >200 participants but results not conclusive.
	Yuan et al., 2008 [86,87]	Non-Cochrane Systematic Review	Chronic low back pain	23	‘There is moderate evidence that acupuncture is more effective than no treatment, and strong evidence of no significant difference between acupuncture and sham acupuncture, for short-term pain relief.’	=	‘… although 16/23 of the studies (70%) scored highly on the Van Tulder scale, only 8/23 had more than 40 patients per group of which 2 studies had high dropouts leaving only 6/23 high quality studies.’	2 studies had trial arms of >200 participants. Both scored 8 or above on the Van Tulder scale. Results are conflicting.
	Furlan et al., 2005 [88]	Cochrane review	Low back pain	35	‘The data do not allow firm conclusions about the effectiveness of acupuncture for acute low-back pain.’	=	‘The methodologic quality of the included RCTs … was poor. There were two studies with fatal flaws …’	One study had trial arms of >200 participants.
	Manheimer et al., 2005 [89]	Non-Cochrane Systematic Review	Chronic low back pain	33	‘Acupuncture effectively relieves chronic low back pain. No evidence suggests that acupuncture is more effective than other active therapies.’	?	No statement on methodological quality was included.	All of the studies included had trial arms of <100 participants.
	Yuan et al., 2004 [90]	Non-Cochrane Systematic Review	Non-specific low back pain	10	‘This review has provided strong evidence that there is no significant difference between acupuncture and sham acupuncture …’	−	‘Ten high-quality studies, with a mean Van Tulder score of 6.6/11, met the inclusion criteria …’	Includes two studies of high quality with trial arms of >200
	Henderson 2002 [91]	Non-Cochrane Systematic Review	Chronic low back pain	5 (11 studies in total)	‘Systematic examination of these articles did not provide definitive evidence to support or refute the use of acupuncture ….’	?	No quality assessment conducted.	One study with n = 262 was inconclusive. Only one other RCT with positive results had n = 28.
	van Tulder et al., 1999 [92]	Cochrane review	Non-specific low back pain	11	‘The evidence … does not indicate that acupuncture is effective for the treatment of back pain.’	?	‘The methodological quality was low. Only two trials were of high quality.’	All studies had small sample sizes of 100 or less.
	Strauss et al., 1999 [93]	Non-Cochrane Systematic Review	Chronic low back pain	4	‘One cannot necessarily conclude from this review whether acupuncture is an effective treatment.’	?	‘… all the trials were of poor quality.’	Minimal information on primary studies included in review including study design and number of patients.
	Ernst & White 1998 [94]	Non-Cochrane Systematic Review	Low back pain	12	‘… insufficient evidence to state whether it is superior to placebo.’	?	‘… Only 2 trials … were of low quality. Thus, the present meta-analysis is based largely on rigorous research.’	All samples sizes were less than 100.
**(b) Mixed**								
	Cherkin et al., 2003 [95]	Non-Cochrane Systematic Review	Acute and chronic back pain	6	‘Because the quality of the research evaluating the effectiveness of the most popular CAM therapies used for low back pain is generally poor, clear conclusions are difficult to reach…’	?	‘The trials had serious limitations, including small sample sizes, inadequate acupuncture treatment, and high dropout rates.’	The largest included study had n = 262. All others were<100.
	van Tulder et al., 1999 [96]	Non-Cochrane Systematic Review	Low back pain (acute and chronic)	11	‘…this systematic review did not clearly indicate that acupuncture is effective in the management of back pain …’	?	‘Overall, the methodologic quality was low. Only two studies met the pre-set “high-quality” level for this review.’	All included studies had sample sizes of <100
**(c) Back and neck Pain**								
	Griswold et al., 2019 [97]	Non-Cochrane Systematic Review	Spine-related painful conditions	12	‘Both superficial and deep needling resulted in clinically meaningful changes in pain scores over time.’	?	‘The included studies demonstrated an unclear to high risk of bias recommending a cautious interpretation of the results.’	This article has a delayed release (embargo) and will be available in 2020
	Yuan et al., 2015 [98]	Non-Cochrane Systematic Review	Chronic neck and low back pain (CNP and CLBP)	30 (48 studies in total)	‘Acupuncture, acupressure, and cupping could be efficacious in treating the pain and disability associated with CNP or CLBP in the immediate term.’	?	‘In summary, many more studies with higher quality and longer-term follow-ups are warranted.’	All trial arms had <200 participants and pooled events <500
	Smith et al., 2000 [99]	Non-Cochrane Systematic Review	Chronic neck and back pain	13	‘There is no convincing evidence for the analgesic efficacy of acupuncture for back or neck pain.’	?	‘With acupuncture for chronic back and neck pain, we found that the most valid trials tended to be negative.’	Trial arms all had <200 participants.
	Ter Riet et al., 1989 [19]	Non-Cochrane Systematic Review	Neck and back pain	16 (22 studies)	‘… it is impossible to draw definite conclusions.’ The authors noted the presence of publication bias.	?	‘The quality was generally low and low therefore no definitive conclusions can be drawn.’	Sample sizes insufficient. One study with trial arm ≥50 participants
**NECK PAIN**								
	Seo et al., 2017 [100]	Non-Cochrane Systematic Review		16	‘Acupuncture and conventional medicine for chronic neck pain have similar effectiveness on pain and disability...’	?	‘…. a lot of the results were evaluated to have low level of evidence, making it difficult to draw clear conclusions …’	Trial arms <200 participants, pooled events < 500.
	Moon et al., 2014 [101]	Non-Cochrane Systematic Review	Whiplash-associated disorder	6	‘In conclusion, the evidence for the effectiveness of acupuncture therapy for whiplash associated disorder is limited.’	?	‘Most of the included RCTs have serious methodological flaws.’	No trials arms with >200 participants
	Wang et al., 2011 [102]	Non-Cochrane Systematic Review	Cervical spondylosis	8	‘At the present, there has been no sufficient evidence to ensure that … abdominal acupuncture therapy is superior …’	?	‘Attention should be paid to the randomized controlled study of larger samples and qualified design.’	Paper in Chinese therefore information taken from abstract. Small sample sizes.
	Fu et al., 2009 [103]	Non-Cochrane Systematic Review		14	‘The quantitative meta-analysis … confirmed the short-term effectiveness and efficacy of acupuncture in the treatment of neck pain.’	+	‘… evidence supporting the main hypothesis that acupuncture was effective in the treatment of neck pain was stronger than the evidence denying this …’	Only one study with sufficient power including >200 participants per trial arm.
	Trinh et al., 2006 [104]	Cochrane review	Neck disorders	10	‘Individuals with chronic neck pain who received acupuncture reported, on average, better pain relief immediately after treatment and in the short-term than those who received sham …’	?	‘… the overall quality of these studies was not considered high, with only 40% of the studies (4/10) considered as high quality …’	None of included studies had arms >200
	White & Ernst 1999 [105]	Non-Cochrane Systematic Review	Neck pain	14	‘… the hypothesis that acupuncture is efficacious … is not based on the available evidence from sound clinical trials.’	?	‘… the methodological quality of the studies, as assessed by the three criteria of the modified Jadad score for clinical trials, was disappointing.’	Included studies all had arms with <100 participants.
**MYOFASCIAL PAIN/TRIGGER POINTS (MTPs)**								
	Espejo-Antúnez et al., 2017 [106]	Non-Cochrane Systematic Review	MTPs	15	‘Our review suggests a short-term positive impact of dry needling on pain intensity and insufficient evidence on the long-term effectiveness, in line with the findings of previous systematic reviews.’	?	‘The 15 randomized controlled trials had a mean method quality score of 7.53 ± 1.30 out of 10, ranging from 5 to 9 in the PEDro scale.’	Dry needling studies. Included studies all had arms with <100 participants.
	Li et al., 2017 [107]	Non-Cochrane Systematic Review	Myofascial Pain syndrome	33	‘… most acupuncture therapies, including acupuncture combined with other therapies, showed superiority over the other single physical therapies …’	?	‘The quality of this analysis is restricted by the quality of the underlying data.’	Included studies all had arms with <100 participants.
	Wang et al., 2017 [108]	Non-Cochrane Systematic Review	Myofascial pain syndrome (MPS)	10	‘… we have demonstrated favourable efficacy of MA in terms of pain relief as well as the reduction of muscle irritability due to MPS when myofascial trigger points (but not acupuncture points) are stimulated …	?	‘High RoB, variable duration of symptoms and differences in the severity of initial conditions may partly influence the validity of the conclusions.’	Included studies all had arms with <100 participants.
	Rodríguez-Mansilla et al., 2016 [109]	Non-Cochrane Systematic Review	Myofascial pain syndrome	10	‘… Dry needling was more effective in decreasing pain comparing to no treatment, it was not significantly different from placebo in decreasing pain.’	?	Authors report that methodological quality was variable from good to poor.	Dry needling studies. Included studies all had arms with <100 participants.
	Cagnie et al., 2015 [110]	Non-Cochrane Systematic Review	Trigger Points the upper trapezius in patients with neck pain	15	‘There is moderate evidence for ischemic compression and strong evidence for dry needling to have a positive effect on pain intensity.’	?	‘Six articles were of low quality and were not further included in the analysis; 9, of moderate quality; and 6, of good quality.’	Dry needling and ischaemic compression. Included studies all had arms with <100 participants.
	Ong et al., 2014 [111]	Non-Cochrane Systematic Review	MTPs in neck and shoulders	5	‘… there is no significant difference between dry needling and lidocaine ….’	?	‘Four out of five RCTs were rated as high-quality (≥6/10); only one RCT rated as low-quality evidence (≤5/10).’	Included studies all had arms with <100 participants. Pooled events < 500
	Kietrys et al., 2013 [112]	Non-Cochrane Systematic Review	Upper quarter myofascial pain	12	‘… we recommend dry needling, compared to sham or placebo, for decreasing pain immediately after treatment and at 4 weeks …’	?	‘… variance in comparison groups, control conditions, dosage of intervention, outcomes, outcome measurement tools, times to outcomes, and internal validity …’	Included studies all had arms with <200 participants.
	Tough & White 2011 [113]	Non-Cochrane Systematic Review	MTP pain	3	‘There is limited evidence that direct MTrP (myofascial trigger points) dry needling has an overall treatment effect when compared with standard care.’	?	‘… there is still a need for large scale, adequately powered, high-quality placebo-controlled trials to provide a more conclusive result.’	Included studies all had arms with <100 participants.
	Cotchett et al., 2010 [114]	Non-Cochrane Systematic Review	MTPs associated with plantar heel pain	3 (non-RCT)	‘There is limited evidence for the effectiveness of dry needling and/or injections of MTrPs associated with plantar heel pain.’	?	‘…the poor quality and heterogeneous nature of the included studies precludes definitive conclusions being made.’	Included studies all had arms with <50 participants.
	Tough et al., 2009 [115]	Non-Cochrane Systematic Review	MTP pain	7	‘… limited evidence deriving from one study that deep needling directly into myofascial trigger points has an overall treatment effect …’	?	‘… the limited sample size and poor quality of these studies highlights and supports the need for large scale, good-quality placebo-controlled trials …’	One study with n = 296. All other studies with small sample sizes.
	Cummings et al., 2001 [116]	Non-Cochrane Systematic Review	MTP pain	23	‘… the hypothesis that needling therapies have efficacy beyond placebo is neither supported nor refuted by the evidence from clinical trials.’	?	‘No trials were of sufficient quality or design to test the efficacy of any needling technique beyond placebo in the treatment of myofascial pain.’	One study with n = 296. All other studies with small sample sizes.
**CANCER PAIN**								
	Chiu et al., 2017 [117]	Non-Cochrane Systematic Review	Malignancy-related, surgery-related or other treatment-related pain.	29	‘Acupuncture is effective in relieving cancer-related pain, particularly malignancy-related and surgery-induced pain.’	?	‘… methodological limitations … affected the strength of evidence and limited the internal validity of this review.’	Included studies all had arms with <100 participants.
	Hu et al., 2016 [118]	Non-Cochrane Systematic Review	Cancer-related pain	20	‘Acupuncture plus drug therapy is more effective than conventional drug therapy alone for cancer-related pain.’	?	‘…GRADE analysis revealed that the quality of all outcomes about acupuncture plus drug therapy was very low.’	Included studies all had arms with <100 participants.
	Paley et al., 2015 [119]	Cochrane review	Cancer pain	5	‘We conclude that there is insufficient evidence to judge whether acupuncture is effective in relieving cancer pain in adults.’	?	‘… the available evidence is of low quality. Therefore, a judgement on whether acupuncture is effective cannot be made.’	Included studies all had arms with <100 participants.
	Lian et al., 2014 [120]	Non-Cochrane Systematic Review	Palliative care symptoms including pain	6 (33 in total)	‘The result … suggested that the effectiveness of acupuncture in palliative care for cancer patients is promising, especially in reducing … cancer pain.’	?	‘Although the RCTs included in this study have relatively high quality, nearly half of them still rated as Jadad score 2 or below.’	Included studies all had arms with <50 participants.
	Garcia et al., 2013 [121]	Non-Cochrane Systematic Review	Cancer care (8 symptoms including pain)	11	‘… appropriate adjunctive treatment for chemotherapy-induced nausea/vomiting … For other symptoms’ management, efficacy remains undetermined.’	?	‘Of the 11 trials examining acupuncture for pain, nine were positive, but eight had high ROB (risk of bias).’	Included studies all had arms with <100 participants.
	Choi et al., 2012 [122]	Non-Cochrane Systematic Review	Cancer pain	15	‘The total number of RCTs included in the analysis and their methodological quality were too low to draw firm conclusions.’	?	‘As suggested by previous systematic reviews … methodological flaws suggest that caution should be taken when interpreting the results of these studies …’	Included studies all had arms with <100 participants.
	Paley et al., 2011 [123]	Cochrane review	Cancer pain	3	‘There is insufficient evidence to judge whether acupuncture is effective in treating cancer pain in adults.’	?	‘Acupuncture is widely used to treat cancer-related pain, but the available evidence is of low quality.’	Included studies all had arms with <100 participants.
	Peng et al, 2010 [124]	Non-Cochrane Systematic Review	Cancer pain	7	‘Acupuncture is effective for pain relief.’	?	‘… the poor quality of the majority of the trials reduces the reliability of the conclusion.’	Article in Chinese. Included studies all had arms with <100 participants.
	Ernst & Lee 2010 [125]	Systematic review of systematic reviews	Palliative and supportive cancer care	7 SR	‘In conclusion, chemotherapy-induced nausea and vomiting is the only indication for acupuncture that is currently supported …’	?	‘… SRs of acupuncture tended to be based on poor-quality primary studies. Our analysis confirms this notion.’	Short report. Only one good-quality SR on cancer pain including 7 primary studies of low quality.
	Lee et al., 2005 [126]	Non-Cochrane Systematic Review	Cancer-related pain	7 (3 RCT)	‘The notion that acupuncture may be an effective analgesic adjunctive method for cancer patients is not supported by the data currently available from the majority of rigorous clinical trials.’	?	‘Due to a dearth of high-quality primary studies in this field, no informative conclusion could be drawn.’	Included studies all had arms with <100 participants.
**FIBROMYALGIA**								
	Zhang et al., 2019 [127]	Non-Cochrane Systematic Review	Fibromyalgia	12	‘… there was moderate quality evidence showing that real acupuncture was more effective than sham acupuncture in the short term.’	?	‘… most of the studies had a relatively small sample size … Second, there was considerable heterogeneity in our meta-analysis.’	Included studies all had arms with <100 participants.
	Kim et al., 2019 [128]	Non-Cochrane Systematic Review	Fibromyalgia	10	‘… verum acupuncture compared with sham acupuncture has a short-term efficacy on reducing pain …’	?	‘… high heterogeneity downgraded the level of evidence.’	Included studies all had arms with <100 participants.
	Yang et al., 2014 [129]	Non-Cochrane Systematic Review	Fibromyalgia	9	‘… there was not enough evidence to prove the efficacy of acupuncture therapy for the treatment of fibromyalgia.’	?	‘… the included trials were not of high quality or had high bias risks.’	Included studies all had arms with <50 participants.
	Cao et al., 2013 [130]	Non-Cochrane Systematic Review	Fibromyalgia	16	‘Acupoint stimulation appears to be effective … compared with medications.’	?	‘The quality of the included studies is generally poor …’	Included studies all had arms with <50 participants.
	Deare et al., 2013 [131]	Cochrane review	Fibromyalgia	9	‘Overall, there is a … moderate level of evidence that acupuncture is not better than sham controls.’	?	‘The small sample size, scarcity of studies for each comparison, lack of an ideal sham acupuncture weakens the level of evidence ….’	Included studies all had arms with <100 participants. Pooled events < 500.
	Cao et al., 2010 [132]	Non-Cochrane Systematic Review	Fibromyalgia	12 (25 studies in total)	‘…. acupuncture was significantly better than conventional medications for reducing pain and number of tender points … significantly better than amitriptyline for preventing relapse.’	?	‘Seven trials (28%) were evaluated as having a low risk of bias and the remaining trials were identified as being as unclear or having a high risk of bias.’	Included studies all had arms with <100 participants.
	Langhorst et al., 2010 [133]	Non-Cochrane Systematic Review	Fibromyalgia	7	‘… small analgesic effect of acupuncture was present … not clearly distinguishable from bias.’	?	‘… great variability of the methodological quality of studies … not robust against potential methodological biases.’	Included studies all had arms with <50 participants.
	Martin-Sanchez et al., 2009 [134]	Non-Cochrane Systematic Review	Fibromyalgia	6	‘This systematic review found no evidence of benefit resulting from acupuncture versus placebo, as a treatment for fibromyalgia.’	?	No specific statement on methodological quality was made but the authors stated there were reporting inconsistencies.	Included studies all had arms with <100 participants.
	Mayhew et al., 2007 [135]	Non-Cochrane Systematic Review	Fibromyalgia	5	‘The notion that acupuncture is an effective symptomatic treatment … is not supported by the results from rigorous clinical trials.’	?	‘… methodological quality was mixed and frequently low.’	Included studies all had arms with <100 participants.
	Berman et al., 1999 [136]	Non-Cochrane Systematic Review	Fibromyalgia (FMS)	3 (7 studies in total)	‘… real acupuncture is more effective than sham acupuncture for improving symptoms of patients with FMS… this conclusion is based on a single high-quality study …’	?	‘… limited amount of high-quality evidence …’	Included studies all had arms with <100 participants. Only one high quality RCT.
**PELVIC PAIN **								
	Qin et al., 2019 [137]	Non-Cochrane Systematic Review	Chronic prostatitis/pelvic pain	6 (4 RCT)	‘Acupuncture may have clinically long-lasting benefits … However, current evidence is limited …’	?	‘… insufficient quantity of studies and small sample size limited to conduct the robust evidence.’	Included studies all had arms with <100 participants.
	Zhang et al., 2019 [138]	Overview of SRs	Primary Dysmenorrhea	5 SRs	‘… there are insufficient qualified evidences to determine the effectiveness of acupuncture in the treatment of PD.’	?	‘All five SRs have more than one critical weakness … their methodological qualities were considered as critically low.’	Ranking of all 5 SRs was ‘critically low’. No information on sample sizes in primary studies.
	Woo et al., 2018 [139]	Non-Cochrane Systematic Review	Primary Dysmenorrhea	60	‘The results of this study suggest that acupuncture might reduce menstrual pain … compared to no treatment or NSAIDs.’	=	‘… the quality of the included RCTs was low, and methodological restriction existed in this study.’	One study with arms including 344/173 patients. Pooled events >500 for MA vs no treatment.
	Sung et al., 2018 [140]	Non-Cochrane Systematic Review	Chronic pelvic pain in women	4	‘The results of our review and meta-analysis suggest the effectiveness of AT (acupuncture)…’	?	‘… most of the included studies had low methodological quality in the Cochrane ROB assessment.’	Included studies all had arms with <200 participants.
	Chang et al., 2017 [141]	Non-Cochrane Systematic Review	Chronic prostatitis/pelvic pain (CP/CPPS)	7	‘Acupuncture has promising efficacy for patients with CP/CPPS. Compared to standard medical treatment, it has better efficacy.’	?	‘The heterogeneous composition … contribute to the heterogeneity and possible effect modification or interactions.’	Included studies all had arms with <100 participants.
	Liu et al., 2017 [142]	Non-Cochrane Systematic Review	Primary Dysmenorrhea (PD)	23	‘The available evidence suggests that acupuncture may be effective for PD and justifies future high-quality studies.’	=	‘… most trials had an unclear or a high risk of bias, which may have caused an overestimation or underestimation of the true treatment effect.’	Two larger studies n = 501 and n = 600 with low risk of bias.
	Xu et al., 2017 [143]	Non-Cochrane Systematic Review	Endometriosis-related pain	10	‘… acupuncture reduces pain and serum CA-125 levels, regardless of the control intervention used.’	?	‘To confirm this finding, additional studies with proper controls, blinding methods, and adequate sample sizes are needed.’	Included studies all had arms with <50 participants.
	Xu et al., 2017 [144]	Non-Cochrane Systematic Review	Primary Dysmenorrhea	16	‘The current evidence reveals that acupoint-stimulation in the treatment of PD has some obvious advantages compared with treatment by NSAIDs.’	?	‘… sample sizes were small, leading to a low inspection efficiency … inadequate reporting of allocation concealment … the results were heterogeneous …’	Included studies all had arms with <100 participants.
	Xu et al., 2014 [145]	Non-Cochrane Systematic Review	Primary Dysmenorrhea	20	‘… acupoint therapy can relieve pain effectively for individuals with PD, and these treatments have advantages in overall efficiency.’	=	‘Insufficient high-quality evidence is available in the current literature …. Hence, the findings … are by no means definitive.’	Study arms <200 but pooled events >500. However, conclusions not definitive due to quality issues
	Chen et al., 2013 [146]	Non-Cochrane Systematic Review	Primary Dysmenorrhea	4	‘… insufficient high-quality evidence available … regarding the effectiveness of acupuncture ….’	?	‘We were only able to determine that one of the acupuncture trials identified was free of selective reporting.’	Only studies using the SP6 acupoint were included. Included studies all had arms with <100 participants.
	Chung et al., 2012 [147]	Non-Cochrane Systematic Review	Primary Dysmenorrhea	30	‘… acupoint stimulation, especially non-invasive acupoint stimulation, could have good short-term effects on the pain of primary dysmenorrhea.’	?	‘… the poor quality of the methodology of the studies was indicated by a low average Jadad score, with 84% … scoring less than 3.’	Included studies all had arms with <200 participants. Most studies had Jadad scores of 1 or 2.
	Cohen et al., 2012 [148]	Non-Cochrane Systematic Review	Chronic prostatitis/chronic pelvic pain	35	‘A statistically significant placebo effect was found for all outcomes and time analysis showed that efficacy of all treatments increased over time.’	?	‘… there was a wide range of study quality. Several trials had questionable placebo groups and inadequate blinding. This makes interpretation of the results difficult …’	Included studies all had arms with <200 participants. Pooled events < 500.
	Posadzki et al., 2012 [149]	Non-Cochrane Systematic Review	Chronic nonbacterial prostatitis + chronic pelvic pain syndrome	9	‘The evidence … syndrome is encouraging but, because of several caveats, not conclusive …’	?	‘… methodologic quality was variable; most were associated with major flaws. Only one RCT had a Jadad score of more than 3…’	Included studies all had arms with <200 participants. Pooled events < 500
	Zhu et al., 2011 [150]	Cochrane review	Endometriosis	1	‘The evidence to support the effectiveness of acupuncture for pain in endometriosis is limited ….’	?	‘The trial included in this review was methodologically weak.’	Only one low-quality RCT with n = 67 included in the review.
	Cho et al., 2010 [151]	Non-Cochrane Systematic Review	Primary Dysmenorrhea	27	‘The review found promising evidence in the form of RCTs for the use of acupuncture ….’	?	‘… the results were limited by methodological flaws.’	Included studies all had arms with <200 participants. Possible publication bias.
	Ee et al., 2008 [152]	Non-Cochrane Systematic Review	Pelvic and back pain in pregnancy	3	‘We conclude that limited evidence supports acupuncture use in treating pregnancy-related pelvic and back pain.’	?	‘Additional high-quality trials are needed to test the existing promising evidence for this relatively safe and popular complementary therapy.’	Based on 3 trials with insufficient sample sizes. Included studies all had arms with <200 participants.
**INFLAMMATORY ARTHRITIS**								
	Ramos et al., 2018 [153]	Overview of Systematic Reviews	Rheumatoid arthritis	7 SR	‘The use of acupuncture probably has minimal or no impact on joint pain in rheumatoid arthritis.’	−	No formal statement of methodological quality was made but the GRADE score for the pain studies was moderate.	20 primary RCTs included. Pain data included from only 2 primary studies.
	Seca et al., 2019 [154]	Non-Cochrane Systematic Review	Rheumatoid arthritis (RA)	13	‘Evidence suggests that acupuncture interventions may have a positive effect in pain relief, physical function and HRQoL (health related quality of life) in RA patients.’	?	‘… due to the heterogeneity and methodologic limitations of the studies included in this systematic review, evidence is not strong enough to produce a best practice guideline.’	Ten studies were published in China. No information available on sample sizes
	Lu et al., 2016 [155]	Non-Cochrane Systematic Review	Gouty arthritis	28	‘… we cautiously suggest that acupuncture is an effective and safe therapy for patients with gouty arthritis.’	?	‘the methodological qualities of included studies were judged to be poor; …’	Included studies all had arms with <100 participants. All were Chinese and single-site studies.
	Lee et al., 2013 [156]	Non-Cochrane Systematic Review	Gouty arthritis	10	‘This study demonstrates efficacy of acupuncture treatment in decreasing VAS and uric acid in gout.’	?	‘… the quality of the trials in this study is generally weak …’	Included studies all had arms with <200 participants. All studies were Chinese.
	Lee et al., 2008 [157]	Non-Cochrane Systematic Review	Rheumatoid arthritis	8	‘… penetrating or non-penetrating sham-controlled RCTs failed to show specific effects of acupuncture for pain control…’	?	‘The number, size and quality of the RCTs are too low to draw firm conclusions.’	Included studies all had arms with <200 participants. Possible publication bias.
	Wang et al., 2008 [158]	Non-Cochrane Systematic Review	Rheumatoid arthritis	8	‘Despite some favourable results in active-controlled trials, conflicting evidence exists in placebo-controlled trials concerning the efficacy of acupuncture for RA.’	?	‘… inappropriate control interventions (non-comparable), no double-blind interventions, inadequate description of the randomization process, and scarce use of validated outcome measures.’	Included studies all had arms with <200 participants. Possible publication bias.
	Casimiro et al., 2005 [159]	Cochrane review	Rheumatoid arthritis	2	‘… electroacupuncture may be beneficial … the reviewers concluded that the poor quality of the trial, including the small sample size preclude its recommendation.’	?	‘… poor quality of the trials, the high methodological variability … and the small sample size of the included studies.’	Only 2 studies met the inclusion criteria. Sample sizes n = 64 and n = 20
	Lautenschlager 1997 [160]	Non-Cochrane Systematic Review	Inflammatory rheumatic diseases	17	‘Acupuncture cannot be recommended for treatment of these diseases.’	?	‘By far, the most studies examined failed to show sufficient quality.’	Written in German. No information about sample sizes.
**NEUROPATHIC PAIN AND NEURALGIA**								
	Pei et al., 2019 [161]	Non-Cochrane Systematic Review	Post-herpetic neuralgia	8	‘… the quality of evidence was low because of the lack of blinding and the small sample sizes of the included studies.’	?	‘… the quality of evidence was moderate for the assessment of pain intensity’.	Seven out of eight studies published in China. Included studies all had arms with <50 participants.
	Hu et al., 2019 [162]	Non-Cochrane Systematic Review	Trigeminal neuralgia	33	‘… no statistically significant differences between the two groups for alleviating pain intensity.’	?	‘… all current evidence is very limited due to the overall low methodological quality of the included RCTs.’	Only 3 small studies included with pain as an outcome measure. Included studies all had arms with <200 participants.
	Oh & Kim 2018 [163]	Non-Cochrane Systematic Review	Chemotherapy-induced peripheral neuropathy	5 (22 studies)	‘… these results provide little evidence of the effectiveness of acupuncture …’	−	Written in Korean. Insufficient high-quality data to make a judgement.	Only 5 included RCTs Acupuncture study arms had <200 participants.
	Choi et al., 2018 [23]	Cochrane review	Carpal tunnel syndrome	12	‘… there is currently insufficient evidence to assess the effectiveness of acupuncture for symptoms of CTS.’	?	‘Most studies were very small (fewer than 100 participants) and all estimates of effects suffered from imprecision.’	Included studies all had arms with <200 participants.
	Wang et al., 2018 [164]	Non-Cochrane Systematic Review	Diabetic peripheral neuropathy	14	‘… ST36 injection appears … effective in reducing pain score and improving NCV compared with intramuscular injection …. ‘	?	‘… poor methodological and reporting quality reduced confidence in the findings.’	Included studies all had arms with <200 participants.
	Wang 2018 [165]	Non-Cochrane Systematic Review	Post-herpetic neuralgia	7	‘… acupuncture is safe and might be effective in pain relieving for patients with PHN.’	?	‘Given the low quality of included studies, the results are not conclusive …’	Included studies all had arms with <200 participants. Pooled events < 500
	Dimitrova et al., 2017 [166]	Non-Cochrane Systematic Review	Peripheral neuropathy	13 (15 studies)	‘This systematic review suggests that acupuncture is effective in diabetic neuropathy, Bell’s palsy, and CTS…’	?	‘… various methodological issues were identified.’	Two studies reported a sample size calculation. Included studies all had arms with <200 participants.
	Ju et al., 2017 [167]	Cochrane review	Neuropathic pain	6	‘… there is insufficient evidence to support or refute the use of acupuncture for neuropathic pain …’	?	‘The overall quality of evidence is very low due to study limitations …’	Included studies all had arms with <200 participants.
	Franconi et al., 2013 [168]	Non-Cochrane Systematic Review	Chemotherapy-induced peripheral neuropathy	3 (6 studies)	‘…although there are some indications that acupuncture may be effective … the current evidence available is limited.’	?	‘All the clinical studies reviewed had important methodological limitations.’	Only 3 studies were RCTs and all had arms with <200 participants.
	Sim, et al., 2011 [169]	Non-Cochrane Systematic Review	Carpal tunnel syndrome	6	‘The existing evidence is not convincing enough to suggest that acupuncture is an effective therapy for CTS.’	?	‘The total number of included RCTs and their methodological quality were low.’	Included studies all had arms with <200 participants.
	Liu et al., 2010 [170]	Non-Cochrane Systematic Review	Trigeminal neuralgia	12	‘The evidence reviewed previously suggests that acupuncture is of similar efficacy as CBZ but with fewer adverse effects …’	?	‘… the evidence is weak because of low methodological quality of the reviewed studies.’	All studies Chinese. Included studies all had arms with <200 participants.
	Longworth et al., 1997 [171]	Non-Cochrane Systematic Review	Sciatica	7 (38 studies in total)	‘The association between acupuncture (AP) and pain relief is so strong that it has tended to obscure any other … clinical results.’	?	‘Although plentiful, the research is variable in quality, especially with respect to design, consistency, and follow-up.’	Included studies all had arms <200 participants.
**OTHER PAIN CONDITIONS**								
	Vier et al., 2019 [172]	Non-Cochrane Systematic Review	Orofacial pain associated with temporo-mandibular joint dysfunction (TMD)	7	‘To date, there is insufficient data to draw strong conclusions about DN for the treatment of orofacial pain associated with TMD.’	?	‘… due the low quality of evidence and high risk of bias of some included studies, larger and low risk of bias trials are needed …’	Language restrictions. Included studies all had arms with <200 participants.
	Kim et al., 2018 [173]	Systematic review of SRs and network meta-analysis	Aromatase inhibitor induced arthralgia	2 (6 in total)	‘Acupuncture … is recommended for AIA with low overall confidence based on the current evidence.’	?	‘… evidence for acupuncture as an effective treatment for AIA was considered low.’	Only 2 small RCTS of acupuncture included in network analysis with total samples of 20 and 22.
	Pan et al., 2018 [174]	Non-Cochrane Systematic Review	Osteoporosis	35	‘This present systematic review indicated that acupuncture could be an effective therapy for treating osteoporosis.’	?	‘… nearly all Chinese studies reported positive results … and all the studies … in this meta-analysis were Chinese trials.	Publication bias. Included studies all had arms with <200 participants.
	Chau et al., 2018 [175]	Non-Cochrane Systematic Review	Shoulder pain (PSP) in stroke survivors	29	‘…. conventional acupuncture and electroacupuncture could be effective treatments for survivors with PSP, with regard to reducing pain ….’	?	‘… the very high potential for bias was prevalent in the included trials. These methodological flaws may have led to biased results in the included trials …’	All trials were conducted in China. Included studies all had arms with <200 participants.
	Luo et al., 2018 [176]	Non-Cochrane Systematic Review	Osteoporosis	9	‘WNA may have beneficial effects on bone mineral density and VAS scores of patients with primary OP.’	?	‘… all included trials were at high risk of bias and of low quality.’	Warm needle acupuncture (WNA). Included studies all had arms with <200 participants.
	Hall et al., 2018 [177]	Non-Cochrane Systematic Review	Upper extremity pain & dysfunction	11	‘There is very low evidence to support the use of TDN (trigger point dry needling) in the shoulder region for treating patients with upper extremity pain or dysfunction.’	?	‘The current evidence supporting TDN for upper extremity pain and dysfunction is very low, and future research is likely to change treatment effect estimates.’	Included studies all had arms with <200 participants.
	Chen et al., 2017 [178]	Non-Cochrane Systematic Review	Aromatase inhibitor induced arthralgia	5	‘… acupuncture treatment significantly reduced Brief Pan Inventory worst pain scores and WOMAC pain scores after 6-8 weeks …’	?	‘… certain trials recruited a relatively small sample size of patients per treatment group … outcomes were reported inconsistently.’	Included studies all had arms with <50 participants.
	Fernandes et al., 2017 [179]	Non-Cochrane Systematic Review	TMJ disorder (TMD)	4	‘… acupuncture treatment appears to relieve the signs and symptoms of pain in myofascial TMD.’	?	‘… the four included studies revealed two studies of good quality and two studies of weak quality.’	Included studies all had arms with <50 participants.
	Thiagarajah 2017 [180]	Non-Cochrane Systematic Review	Plantar fasciitis	4	‘… acupuncture may reduce … pain in the short term … insufficient evidence for a definitive conclusion regarding … the longer term.’	?	‘The number of participants (range 23–53) was small in all studies and the types of controls employed varied.’	Included studies all had arms with <50 participants.
	Lee & Lim 2016 [181]	Non-Cochrane Systematic Review	Post-stroke shoulder pain	12	‘Although there is some evidence for an effect of acupuncture on poststroke shoulder pain, the results are inconclusive.’	?	‘… some of the included studies were of poor quality and had methodological shortcomings ….’	Included studies all had arms with <100 participants.
	Wang et al., 2016 [182]	Non-Cochrane Systematic Review	Shoulder pain	9	‘Ashi point stimulation might be superior to conventional acupuncture, drug therapy and no treatment for shoulder pain.’	?	‘… most of the trials suffer from many flaws …. Eight out of 9 included studies had severe methodological defects.’	Included studies all had arms with <100 participants.
	Tang et al., 2015 [183]	Non-Cochrane Systematic Review	Lateral epicondylitis	4	‘For the small number of included studies… no firm conclusion can be drawn regarding the effect of acupuncture …’	?	‘The overall quality rated by GRADE was from very low to low.’	Pain was not an outcome measure. Included studies all had arms with <100 participants.
	Chang et al., 2014 [184]	Non-Cochrane Systematic Review	Lateral epicondylitis	9	‘Manual acupuncture is effective in short-term pain relief … however, its long-term analgesic effect is unremarkable.’	−	The analgesic effect of manual acupuncture on the treatment of lateral epicondylalgia is Level B	Included studies all had arms with <100 participants.
	Lee et al., 2012 [185]	Non-Cochrane Systematic Review	Post-stroke shoulder pain	7	‘It is concluded from this systematic review that acupuncture combined with exercise is effective for shoulder pain after stroke.’	?	‘… there were insufficient quality assessments with respect to allocation concealment, blinding of outcome assessors, and long-term follow-up.’	All studies were Chinese. Included studies all had arms with <100 participants.
	Clark et al., 2012 [186]	Non-Cochrane Systematic Review	Plantar heel pain	5 (8 in total)	‘In view of the heterogeneity of these papers, it is not possible to give a simple conclusion ….’	?	‘Two studies provide good reporting of high-quality studies; six are of lesser quality.’	The included RCTs all had arms with <100 participants.
	Smith, et al., 2011 [187]	Cochrane review	Labour pain	13	‘There are insufficient data to demonstrate whether acupuncture and acupressure are more effective than a placebo control, or whether there is additional benefit from acupuncture when used in combination with usual care.’	?	‘The risk of bias was high in the majority of trials and recommendations for practice cannot be made until further high-quality research has been undertaken.’	One large study with >200 participants in the acupuncture group and >100 in the other two groups. Other studies had <200 participants in each arm and relatively high risk of bias.
	Cho et al., 2010 [188]	Non-Cochrane Systematic Review	Labour pain	10	‘The evidence from RCTs does not support the use of acupuncture for controlling labour pain.’	?	‘The primary studies are diverse and often flawed.’	As above—One large study. Others had <200 participants per arm
	La Touche et a, 2010 [189]	Non-Cochrane Systematic Review	TMJ disorder	9	‘… acupuncture is a reasonable adjunctive treatment for producing a short-term analgesic effect ….’	?	‘… the relevance of these results was limited by the fact that substantial bias was present.’	Included studies all had arms with <100 participants.
	Fink et al., 2006 [190]	Non-Cochrane Systematic Review	TMJ disorder	6	‘Acupuncture appears to be a suitable complementary treatment method in the management ….’	?	‘… results achieved must be interpreted with caution because of the methodological shortcomings identified.’	Only 3 electronic databases searched.
	Green et al., 2005 [191]	Cochrane review	Shoulder pain	9	‘There is little evidence to support or refute the use of acupuncture …’	?	‘This review has highlighted the paucity of methodologically rigorous, well described randomised controlled trials with adequate sample size …’	Largest study, with unclear risk of bias, had 150 participants. All others had <200 participants per arm.
	Trinh et al., 2004 [192]	Non-Cochrane Systematic Review	Lateral epicondyle pain	6	‘There is strong evidence suggesting that acupuncture is effective in the short-term relief of lateral epicondyle pain.’	?	‘The six studies being assessed were considered consistent high-quality … because they were all within the 3–5 range on the Jadad scale.’	In spite of the relatively high Jadad score the studies all had small sample sizes, ranging from 17–82.
	Lee & Ernst 2004 [193]	Non-Cochrane Systematic Review	Labour pain management	3	‘Overall, the evidence of acupuncture for pain management during labour is encouraging …’	?	‘The methodologic quality of the primary studies is generally good …’	Conclusions based on 3 studies with trials arms <200 participants.
	Green et al., 2002 [194]	Cochrane review	Lateral elbow pain	4	‘There is insufficient evidence to either support or refute the use of acupuncture …’	?	‘Due to … problems with methodology of the included trials … the results of this review are inconclusive.’	Included studies all had arms with <100 participants.
	Rosted 2001 [195]	Non-Cochrane Systematic Review	TMJ disorder	3	‘Acupuncture has in three out of three randomised controlled trials (RCT) proved effective for the treatment of TMD.’	?	‘… publications … fulfilled the list of predefined methodological criteria with a score between 77% and 84%.’	Trial arms included <100 participants. However, the purpose of this review was to present standard acupuncture procedure.
	Ernst & White 1999 [196]	Non-Cochrane Systematic Review	TMJ dysfunction	3	‘Even though all studies are in accordance with the notion that acupuncture is effective for temporomandibular joint dysfunction, this hypothesis requires confirmation …’	?	‘None of the trials was performed with blinded evaluators, details of randomization are not given, and therefore all studies are subject to important bias.’	Studies as in Rosted 2001 above. All 3 studies from Scandinavia.
	Rosted 1998 [197]	Non-Cochrane Systematic Review	Dentistry	15	‘Acupuncture in 11 out of 15 studies proved effective … as analgesia.’	?	No definitive statement on quality but authors report that 6 studies were of ‘excellent’ or ‘good’ quality.	Insufficient sample sizes.
	Ter Riet et al., 1989 [21]	Non-Cochrane Systematic Review	Facial pain	2	‘The effectiveness of acupuncture on facial pain may not be accepted as proven.’	?	‘The shortcomings of the studies are clear from the table. The big spread in the score of the study [by] Lewith et al. is mainly due to the poor reporting.’	Only two small RCTs included.

Key: * Systematic reviewers’ conclusion about efficacy: Direct quote taken from the conclusion of the article (from either Abstract or Discussion section). ** Our judgement of efficacy within the review: Determined by the following criteria: + means sufficient evidence and in favour of acupuncture; − means sufficient evidence in favour of control/placebo; = means sufficient evidence but inconclusive; ? means insufficient evidence to make a judgement. Sufficient evidence = pooled analysis of 500 events or >200 participants in each arm of at least one RCT. *** Systematic reviewers’ conclusion of quality of available evidence: Direct quote taken from the conclusion of the article (from either Abstract or Discussion section).
